# Meningitic *Escherichia coli* K1 Penetration and Neutrophil Transmigration Across the Blood–Brain Barrier are Modulated by Alpha7 Nicotinic Receptor

**DOI:** 10.1371/journal.pone.0025016

**Published:** 2011-09-22

**Authors:** Feng Chi, Lin Wang, Xueye Zheng, Chun-Hua Wu, Ambrose Jong, Michael A. Sheard, Wei Shi, Sheng-He Huang

**Affiliations:** 1 Department of Pediatrics, Saban Research Institute, University of Southern California, Childrens Hospital Los Angeles, Los Angeles, California, United States of America; 2 Department of Histology and Embryology, School of Basic Medical Science, Wuhan University, Wuhan, China; Louisiana State University, United States of America

## Abstract

Alpha7 nicotinic acetylcholine receptor (nAChR), an essential regulator of inflammation, is abundantly expressed in hippocampal neurons, which are vulnerable to bacterial meningitis. However, it is unknown whether α7 nAChR contributes to the regulation of these events. In this report, an aggravating role of α7 nAChR in host defense against meningitic *E. coli* infection was demonstrated by using α7-deficient (α7^-/-^) mouse brain microvascular endothelial cells (BMEC) and animal model systems. As shown in our *in vitro* and *in vivo* studies, *E. coli* K1 invasion and polymorphonuclear neutrophil (PMN) transmigration across the blood-brain barrier (BBB) were significantly reduced in α7^-/-^ BMEC and α7^-/-^ mice. Stimulation by nicotine was abolished in the α7^-/-^ cells and animals. The same blocking effect was achieved by methyllycaconitine (α7 antagonist). The tight junction molecules occludin and ZO-1 were significantly reduced in the brain cortex of wildtype mice infected with *E. coli* and treated with nicotine, compared to α7^-/-^ cells and animals. Decreased neuronal injury in the hippocampal dentate gyrus was observed in α7^-/-^ mice with meningitis. Proinflammatory cytokines (IL-1β, IL-6, TNFα, MCP-1, MIP-1alpha, and RANTES) and adhesion molecules (CD44 and ICAM-1) were significantly reduced in the cerebrospinal fluids of the α7^-/-^ mice with *E. coli* meningitis. Furthermore, α7 nAChR is the major calcium channel for nicotine- and *E. coli* K1-increased intracellular calcium concentrations of mouse BMEC. Taken together, our data suggest that α7 nAChR plays a detrimental role in the host defense against meningitic infection by modulation of pathogen invasion, PMN recruitment, calcium signaling and neuronal inflammation.

## Introduction

Pathogen penetration and polymorphonuclear neutrophil (PMN) transmigration across the blood-brain barrier (BBB) are the hallmark features of bacterial meningitis, which is the most common serious infection of the central nervous system (CNS) [1]-[2]. For disease to develop, blood-borne pathogens must interact with and penetrate across brain microvascular endothelial cells (BMEC), which form the main constituents of the BBB, and then gain access to the brain and meninges. An overwhelming host inflammatory response, including transendothelial migration of PMN, is provoked upon bacterial internalization and replication within the CNS. While various bacterial determinants and CNS factors that contribute to pathogen invasion, neuronal inflammation and brain injury have been identified and characterized in both *in vitro* and *in vivo* models of bacterial meningitis, little is known about the specific contribution of α7 nAChR, an essential regulator of inflammation, to the pathogenesis of bacterial meningitis.

Bacterial meningitis most frequently results from the bacteremia, which is essential for pathogen invasion across the BBB [1]. There are two important aspects suggested in the gap between the biology of α7 nAChR and bacterial penetration across the BBB. On one hand, an important connection between the nervous system and the inflammatory response to disease has been uncovered through identification of α7 nAChR as an essential regulator of inflammation. As reported by Wang et al., the α7 subunit is essential for inhibiting endotoxin-induced cytokine synthesis in macrophages through the cholinergic anti-inflammatory pathway [3]. Recent studies demonstrated that α7 nAChR played a detrimental role in the host defense against *E. coli* peritonitis and pneumococcal pneumonia [4]–[5]. The host defense against bacterial infection is impaired by stimulation of α7 nAChR with nicotine, which is an α7 agonist derived from tobacco smoke with multiple effects on the vascular, immune and nervous systems [5]-[6]. It is likely that nicotine is able to modulate the host defense system through nAChRs on cells in the tissue barriers, the immune system and the CNS similar to opiates and cannabinoids [7]. We have previously shown that nicotine was able to enhance meningitic *E. coli* K1 invasion of human BMEC *in vitro*, suggesting the involvement of α7 nAChR in the pathogenesis of bacterial meningitis [8]. Although a number of the epidemiological studies have shown that exposure to passive tobacco smoke significantly increases the risk of bacteremia and bacterial meningitis [9]-[11], the pathogenic mechanisms of nicotine and tobacco smoke on this disease are largely unknown. This receptor is abundantly expressed in hippocampus, which is the region most vulnerable to bacterial meningitis. A coordinated response has been demonstrated between α7 nAChR and NMDA receptor (NMDAR) [12]. Excitotoxic neuronal injury by the activity of NMDAR has been implicated in the pathogenesis of bacterial meningitis [13]–[14]. Opposite effects on neonatal excitotoxic brain injuries could be induced by activation or suppression of α7 nAChR in the CNS when compared to that in adults [15], suggesting that meningitic inflammation in neonates and adults may be differentially regulated by nAChRs. On the other hand, α7 nAChR is a member of a family of ligand-gated ion channel, having one of the highest permeabilities to calcium [16]. Cytoplasmic calcium signals are mediated by activation of nAChRs through three different approaches: (a) direct calcium influx through nAChRs, (b) indirect calcium influx through voltage-dependent calcium channels, and (c) calcium-induced calcium release from the endoplasmic reticulum [16]. Regulation of intracellular calcium by α7 nAChR can lead to activation of signal transduction pathways, including extracellular signal-regulated kinase 1/2 (ERK1/2), cAMP response element binding (CREB), and AKT [17]. It has been shown that nicotine was able to activate Calcium/calmodulin-dependent kinase II (CaMKII) in rat prefrontal cortex nerve terminals through α7 nAChR [18]. The prion protein (PrP^c^) could bind to α7 nAChR to form a signaling complex, which led to an increase in intracellular calcium and activation of ERK1/2 [19].

Ca^2+^ signaling has been found to be important in various steps of microbial infection, including meningitis. Bacterial pathogens and their products can induce an increase in intracellular Ca^2+^ in host cells [20]. Pneumolysin, a toxin of meningitic Pneumococcus, was able to induce increases of intracellular Ca^2+^ and trigger brain cell apoptosis [21]. Meningitic *E. coli* was also able to increase cytosolic-free-calcium levels of human BMEC in a manner dependent on calmodulin [22], suggesting that calcium signaling contributes to the pathogenesis of *E. coli* meningitis. Our recent study demonstrated that IbeA (*i*nvasion of *b*rain *e*ndothelium) + *E. coli* invasion of HBMEC was positively correlated with phosphorylation of the IbeA receptor vimentin at Ser82 by CaMKII and pathogen-induced phosphorylation of ERK1/2 [23]. Interaction between IbeA and vimentin at HBMEC membrane rafts is essential for ERK1/2-mediated signalling, which modulate meningitic *E. coli* K1 invasion. Erk1/2 activation is also required for nicotine-enhanced *E. coli* K1 invasion of HBMEC in a manner dependent on the recruitment of α7 nAChR and related signaling molecules, including vimentin, and Erk1/2, to caveolin-1 enriched lipid rafts [24]. It remains to be determined, however, whether and how α7 nAChR-mediated calcium signaling contributes to meningitic invasion *in vitro* and *in vivo*. Therefore, it is important to further dissect its role in the pathogenesis of bacterial meningitis and CNS injury by defining the mechanism by which it modulates pathogen penetration across the BBB.

The migration of leukocytes across the BBB into the CNS is critical in the pathogenesis of bacterial meningitis [25]. It is a key aspect of the protective response against invading pathogens, but in recent years, evidence has accumulated that leukocytes also contribute importantly to the deleterious effects of inflammation on the brain in bacterial meningitis [26]. The adhesive interactions between transmigrating leukocytes and endothelial cells are well understood. We have recently defined *E. coli* K1-induced adhesive interactions between transmigrating leukocytes and brain endothelial cells in a manner dependent on the IbeA receptor vimentin [27]. ICAM-1 and CD44 play a role in the leukocyte transmigration process during *E. coli* meningitis. It has been demonstrated that leukocytes are able to transmigrate across the endothelium by using both paracellular and transcellular pathways. Recent studies show that blood lymphocytes and neutrophils preferentially transmigrate across peripheral and brain endothelial cells via a transcellular route [28]. This notion is supported by our recent findings that transcellular migration of PMN across HBMEC is induced by meningitic *E. coli* K1 [27]. It has been shown that nicotine could induce significant dose-related increases in leukocyte rolling and adhesion in the cerebral microcirculation of the mouse brain [29]. Endothelial cell activation and leukocyte recruitment was regulated through the α7 nAChR cholinergic pathway during endotoxin-induced inflammation [30]. Currently, it is unclear whether and how the α7 nAChR cholinergic pathway contributes to PMN transmigration across the BBB during meningitic infection. As α7 nAChR is a key regulator of inflammation [3], it is important to examine whether this receptor on both leukocytes and the endothelium is essential for modulation of meningitic virulence factor-induced PMN transmigration across the BBB.

In this report, using α7-deficient mouse cell culture and animal model systems, we examined how α7 nAChR contributed to the modulation of pathogen invasion, PMN recruitment and neuronal inflammation induced by *E. coli* K1, which is the most common gram-negative pathogen causing neonatal bacterial meningitis. The *in vitro* and *in vivo* models permit genetic dissection of the role of α7 nAChR in modulation of host defense against meningitic pathogen invasion. We also sought to examine whether the α7 receptor on both BMEC and leukocytes is required for the recruitment of PMN into the CNS, which is associated with increased permeability of the BBB and neuronal injury. Finally, we assessed α7 nAChR-mediated calcium signaling and proinflammatory factors that have the potential to affect the outcome of bacterial meningitis.

## Results

### Isolation and characterization of mouse BMEC (MBMEC) from wildtype and α7 knockout animals

In order to establish *in vitro* models for examining the role of α7 nAChR in *E. coli* invasion and PMN transmigration, wildtype (WT) and α7 nAChR knockout (KO) MBMEC were isolated and purified from the brains of 10-day-old wildtype (α7^+/+^) and α7-deficient mice (α7^-/-^) using UEA I lectin-coated beads as described in Methods and Materials. Under the light microscope, the isolated cells showed endothelial cell type morphology in both the WT and KO MBMEC (Figure S1A). Then, the cells were stained with antibodies against the mouse endothelial marker CD146 (FITC conjugate) and the brain cell markers GGT (FITC) and S100B (FITC), respectively, demonstrating that the cells were derived from brain microvasculature (Figure S1A). The tight junction (TJ) formation was stained with the TJ marker ZO-1 (FITC) (Figure S1A). Next, the deficiency of α7 nAChR was confirmed by the absence of α-bungarotoxin (α-BTX) binding sites in KO MBMEC (Figure S1A) and the KO mouse brain tissues (Figure S1C) using the rhodamine conjugated α-BTX binding assay [31], and the lack of α7 nAChR in KO MBMEC by immunnoblotting with a rabbit antibody against the mouse α7 receptor (Figure S1B). These results confirmed that the α7 nAchR was completely deleted in MBMEC derived from the knockout mice.

### α7-deficient MBMEC are defective in *E. coli* K1 invasion

**Figure 1:**
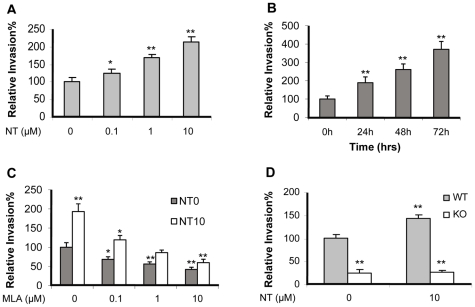
Effects of chemical and genetic blockages of α7 nAChR on E44 invasion *in vitro*. E44 invasion of WT MBMEC after exposure to nicotine (NT) at different doses (0.1 to 10 µM) for 48 h (**A**) and 10 µM of NT at different points (0–72 h) (**B**). (**C**) Effects of different doses of MLA (1 h incubation) on E44 invasion of WT MBMEC treated with (10 µM NT for 48h) and without NT. (**D**) Effect of genetic blockage of α7 on E44 invasion of MBMEC with or without NT treatment (10 µM for 48h). In all treatments, the WT MBMEC without any treatment was taken as a control, and all results are expressed as relative invasion compared the corresponding controls without treatments (100%). All invasion assays were performed in triplicate wells. Bar graphs show the means ± SD of triplicate samples. Significant differences with regard to the controls are marked by asterisks (*P<0.05; **P<0.01).

To determine the role of α7 nAchR in the pathogenesis of *E. coli* meningitis, we examined whether KO MBMEC treated with and without nicotine were defective in bacterial invasion. To mimic the concentrations of nicotine measured in the serum of human active and passive smokers [32], MBMEC (α7^+/+^) were exposed to low doses of nicotine (0.1 to 10 µM) for 48 h or 10 µM of nicotine at different time points (0–72 h). The results indicated that *E. coli* K1 invasion was significantly enhanced by nicotine in a dose- and time-dependent manner (Figure 1A-B). WT MBMEC were then incubated with or without nicotine (10 µM) for 48 hours, and treated with the α7 antagonist methyllycaconitine (MLA). The result indicated that MLA was able to block *E. coli* invasion of MBMEC treated with and without nicotine in a dose-dependent manner (Figure 1C). The WT and KO MBMEC treated with or without nicotine were then subjected to bacterial invasion assays. The invasion rates of WT MBMEC were much higher than that of KO MBMEC even without nicotine stimulation, suggesting that α7 nAChR might play a regulatory role in bacterial invasion in a manner independent of nicotine (Figure 1D). Since nicotine could not increase the invasion rate in KO MBMEC when compared to that in WT MBMEC, α7 nAChR should be the major receptor for nicotine-induced cellular effects. Taken together, these studies suggest that α7 nAChR contributes to bacterial invasion in a nicotine-dependent and independent manner.

### α7 nAChR in BMEC and PMN is required for leukocyte transmigration across MBMEC

**Figure 2:**
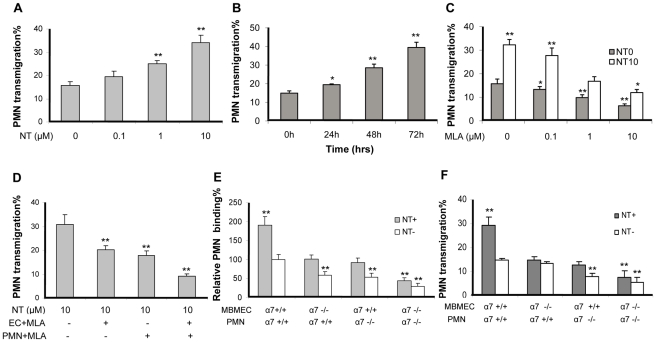
Effects of chemical and genetic blockages of α7 nAChR on NT-enhanced PMN transmigration across BBB. (**A**) E44-induced PMN transmigration across WT MBMEC that were exposed to different doses of NT (0.1 to 10 µM) for 48 h. (**B**) E44-induced PMN transmigration across WT MBMEC that were exposed to NT (10 µM) for 0-72h. (**C**) Effects of different doses of MLA (1 h incubation) PMN transmigration across WT MBMEC treated with (10 µM NT for 48h) and without NT. (**D**) Effect of MLA treatment of either MBMEC or PMN on NT-enhanced PMN transmigration. WT MBMEC were pre-exposed to 10 µM NT for 48 h, and then MBMEC and PMN were treated with MLA for 1 hr prior to the leukocyte transmigration assay. The α7 deficiency of either MBMEC or PMN resulted in a significant suppression of E44-induced PMN binding (**E**) and transmigration (**F**) with or without NT exposure. WT and KO MBMEC were exposed to 10 µM NT for 48 h before the PMN adhesion and transmigration assays. For the PMN adhesion assay, results were expressed as relative adhesion compared to the WT cells (PMN and MBMEC) (100%). Values represent the means of fifteen randomly selected fields from triplicate wells as described in Methods and Materials. For the PMN transmigration assay, values represent the means of % transmigrating PMN of triplicate samples. Bar graphs show the means ± SD of triplicate samples. In (D), the experimental setting without MLA treatment was taken as a control (the first column). In (E) and (F), the WT cells (PMN and MBMEC) without any treatment served as controls. Bar graphs showed the means ± SD of the triplicate samples. *P<0.05, **P<0.01.

PMN recruitment into the CNS plays a crucial role in the inflammatory response during bacterial meningitis [25]. In order to exclude the possibility that the leukocyte migration elicited was due to destruction of MBMEC, the integrity of the monolayer was inspected by microscopy. WT MBMEC were exposed to low doses of nicotine (0.1 to 10 µM) for 48 h or 10 µM of nicotine at different time points (0–72 h), and subjected to PMN transmigration assays. As indicated in Figure 2A and 2B, nicotine significantly increased PMN transmigration in a dose- and time-dependent manner. MLA was able to significantly inhibit PMN transmigration across the wildtype MBMEC monolayer treated with and without nicotine in a dose-dependent manner (Figure 2C). MLA-mediated blocking effects were observed upon treatment of either cell type alone or both (Figure 2D), suggesting that α7 nAChR expression on both leukocytes and MBMEC is required for nicotine-enhanced PMN transmigration *in vitro*. To further support this conclusion, α7^+/+^ and α7^-/-^ MBMEC and PMN were used in leukocyte adhesion and migration assays. As shown in Figure 2E and 2F, both α7^-/-^ MBMEC and α7^-/-^ PMN were significantly defective in leukocyte adhesion and transmigration when compared to the wildtype cells. These results were consisted with the result of chemical blockage by MLA, suggesting that α7 nAChR on BMEC and PMN is required for leukocyte adhesion and transmigration.

### α7 knockout neonatal mice are defective in *E. coli* K1-induced bacteremia, bacterial meningitis, PMN recruitment and nicotine-mediated stimulation

**Figure 3:**
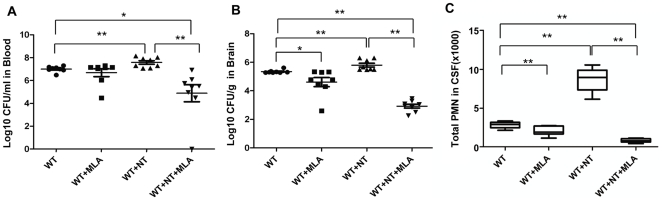
MLA-mediated inhibition of bacteremia, bacterial entry into the brain, and PMN transmigration across BBB. (**A**) Magnitude of bacteremia in WT mice treated with NT or MLA. (**B**) Bacterial loads in the brains of WT mice treated with NT or MLA. (**C**) Migration of PMN into the CSF of WT mice treated with NT or MLA. WT neonatal mice were divided into 4 groups (6–8 pups/group). Each experiment was repeated three times. *P<0.05, **P<0.01.

**Figure 4:**
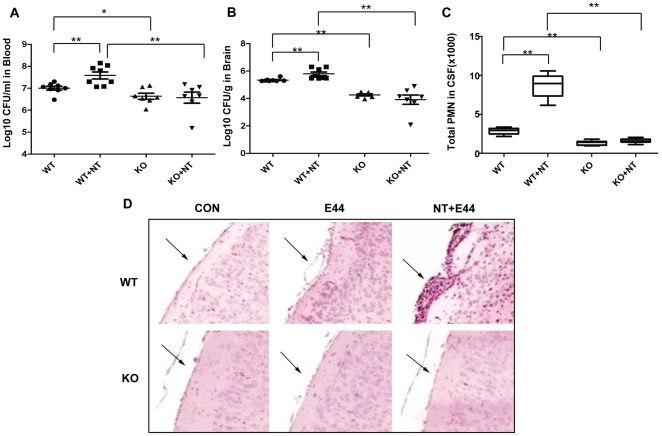
Effects of α7 deficiency on bacteremia, bacterial entry into brain, and PMN transmigration across BBB. (**A**) Magnitude of bacteremia in WT and KO mice treated with or without NT. (**B**) Bacterial loads in the brains of WT and KO mice treated with or without NT. (**C**) Migration of PMN into the CSF of WT and KO mice treated with or without NT. WT and KO neonatal mice were divided into 4 groups (6–8 pups/group). Each experiment was performed three times. *P<0.05, **P<0.01. (**D**) The recruitment of PMN into the CNS of WT and KO mice treated with NT and infected with E44. Brain cortex sections were stained with hematoxylin-eosin. Arrows indicate infiltrating PMN. Images are 200×.

To further validate the biological relevance of the *in vitro* assays, the role of α7 nAChR in the pathogenesis of neonatal *E. coli* K1 meningitis was tested in the mouse model, as described in Methods and Materials. We first examined the effects of the α7 antagonist MLA on nicotine-enhanced meningitis in wildtype mice. In this study, wildtype neonatal (10 day-old) mice were intraperitoneally injected with E44 (2×10^5^ CFU) after treatment with nicotine or MLA for 3 days. As shown in Figure 3, nicotine was able to significantly increase *E. coli* bacteremia (P<0.01, Figure 3A), bacterial entry into brain and CSF (meningitis) (P<0.01, Figure 3B and Figure S2A), and PMN transmigration across the BBB (P<0.01, Figure 3C). MLA was able to significantly block nicotine-enhanced pathogenicities when compared to the controls. These results suggest that α7 nAChR could increase the host susceptibility to *E. coli* K1 meningitis. To further confirm this conclusion, the host susceptibility to *E. coli* K1 meningitis was tested in wildtype and KO neonatal mice. Animals of the same age were intraperitoneally injected with E44 (2×10^5^ CFU), followed by Evans blue injection after 15h. As shown in Figure 4A, bacteremia was significantly decreased in KO mice as compared to wildtype animals (P<0.05), suggesting that α7 nAChR plays a role in the genesis of bacteremia. This result showed that the magnitude of bacteremia was significantly increased by nicotine exposure only in wildtype mice (P<0.01), but not in KO mice, suggesting that α7 nAChR is essential for nicotine-enhanced bacterial pathogenicities (Figure 4A). Similarly, the bacterial counts in brain and CSF were significantly reduced in KO mice as compared to wildtype animals (P<0.01), suggesting that α7 nAChR also contributes to *E. coli* K1 penetration across the BBB (Figure 4B and Figure S2B). *E. coli* K1 was also able to significantly increase PMN transmigration across the BBB into CSF in wildtype mice as compared to KO animals (P<0.01, Figure 4C). Nicotine was only able to enhance PMN transmigration across the BBB in wildtype mice as compared to corresponding controls (P<0.01), suggesting that leukocyte transmigration across the BBB is mainly modulated by α7 nAChR. Histologic examination of brains with hematoxylin-eosin staining indicated that nicotine was able to significantly enhance the recruitment of PMN into the CNS induced by E44 cells in the wildtype mice but not in KO mice (Figure 4D), which further confirmed the role of α7 nAChR in PMN transmigration across the BBB. Taken together, these data suggested that α7 nAChR could play a detrimental role in the host defense against *E. coli* meningitis by increasing *E. coli* bacteremia, bacterial invasion, and PMN transmigration across the BBB.

### *E. coli* bacteremia and meningitis in heterozygous mice

To further examine whether a single allele was sufficient to complement the role of α7 nAchR in the pathogenesis of *E. coli* meningitis, the heterozygous (+/-) neonatal mice were also subjected to i.p. injection of the same inoculum size of E44. The wildtype and heterozygous animals did not show marked differences in bacteremia (Figure S3A), bacterial counts in brain tissues and CSF (Figure S3B and S3C), and the rate of PMN transmigration across the BBB (Figure S3D). These results suggest that a single allele could retain the full function of α7 nAChR to increase host susceptibility to *E. coli* K1 meningitis.

### Tobacco smoking increased *E. coli* K1-induced bacteremia, bacterial meningitis, PMN recruitment into the CNS of neonatal mice

Since nicotine is a major component in tobacco smoke, we examined the effect of tobacco smoking on pathogenesis of neonatal *E. coli* K1 meningitis. Side stream (95%) tobacco smoking was performed from postnatal day 4 to day 10 with wildtype neonatal mice as described in Methods and Materials. At day 10, the neonatal mice with or without tobacco smoking were intraperitoneally injected with E44 (2×10^5^ CFU). As shown in Figure S4, tobacco smoking was able to significantly increase *E. coli* bacteremia (P<0.01, S Figure S4A), bacterial entry into brain tissues and CSF (meningitis) (P<0.01, Figure S4B and S4C), and PMN transmigration across the BBB (P<0.01, Figure S4D). These data suggested that second hand tobacco smoking could be a significant risk factor for *E. coli* meningitis in neonates.

### α7 deficient BMEC and animals are defective in *E. coli* K1- and nicotine-induced BBB disorders

**Figure 5:**
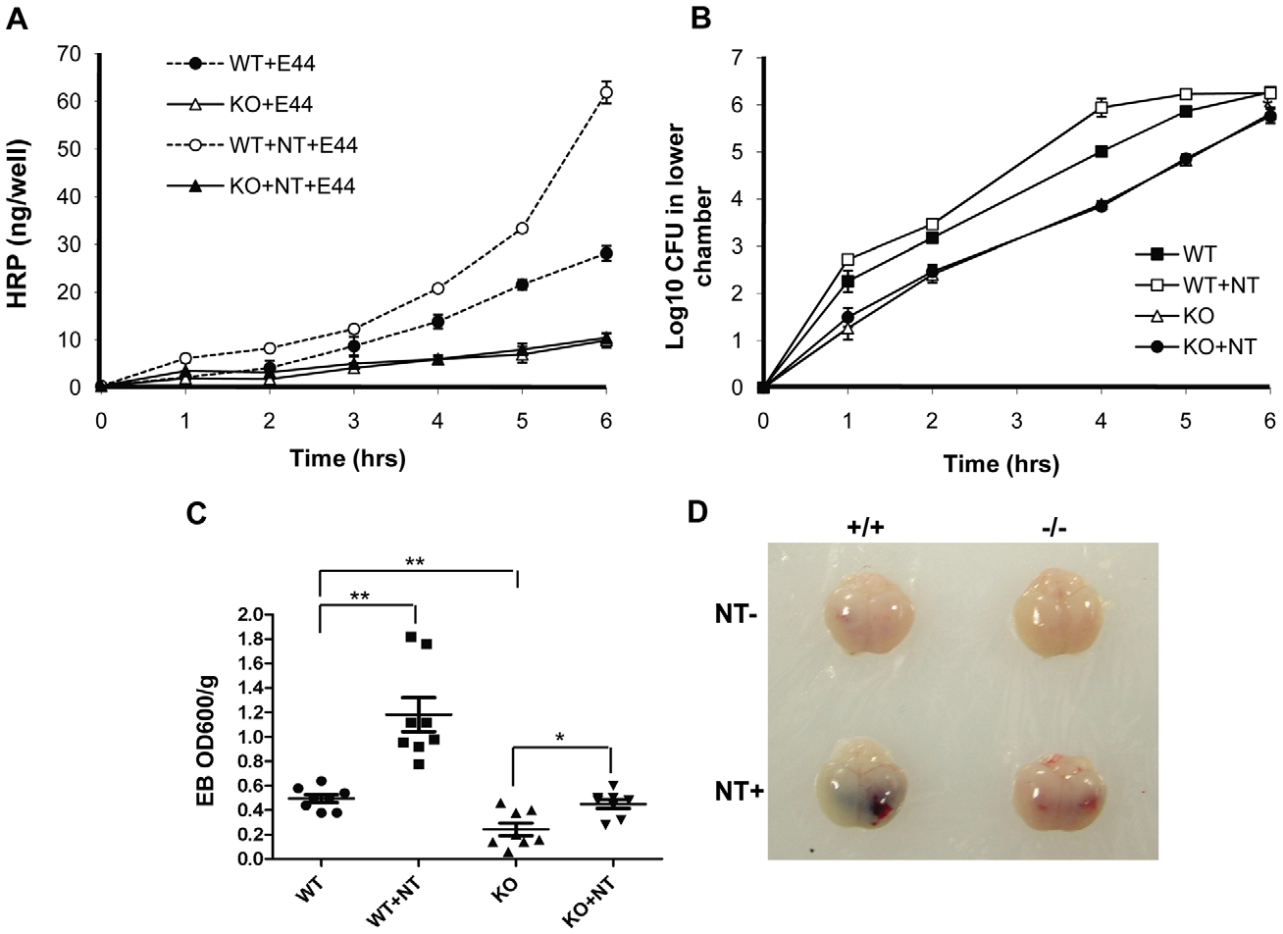
Effects of genetic blockage of α7 nAChR on NT-increased BBB permeability and E44 transcytosis. (**A**) Time-course study of BBB permeability to HRP in WT and KO MBMEC with or without NT (10 µM) exposure for 48 hours. (**B**) Time-course examination of *E. coli* K1 penetration across WT and KO MBMEC treated with or without NT (10 µM). In both (**A**) and (**B**), values represent the means of triplicate samples from lower chambers. (**C**) Evaluation of BBB permeability to Evans blue in WT and KO mice with or without NT exposure (n = 6-8). *P<0.05, **P<0.01. **(D)** Images of mouse brains stained with Evans blue.

As pathogen penetration and PMN transmigration across the BBB are the most critical step in the pathogenesis of bacterial meningitis [1]-[2], we tested whether the BBB permeability was increased by α7 nAChR. BBB permeability was first examined in a Transwell system with wildtype and KO MBMEC treated with nicotine or E44 cells. As shown in Figure 5A, the passage of horseradish peroxidase (HRP) through wildtype MBMEC monolayers was increased upon infection with E44 cells in a time-dependent manner. The E44-mediated stimulation was amplified by exposure to nicotine in the same manner. These results demonstrated that nicotine could enhance the BBB permeability *in vitro*. Nicotine exposure was unable to increase the E44-induced BBB permeability in KO MBMEC as compared to the negative control without nicotine treatment, suggesting that α7 nAChR contributes to increased BBB permeability induced by both *E. coli* K1 and nicotine. *E. coli* K1 translocation across the MBMEC monolayer in the two chamber transwell system was also examined by plating bacteria at different time points (Figure 5B). The result indicated nicotine treatment could accelerate *E. coli* K1 translocation across WT MBMEC, while the α7 nAChR deficiency led to decreases in *E. coli* K1 translocation. These *in vitro* results were consisted with the conclusion drawn from the *in vivo* studies using the mouse model of neonatal *E. coli* meningitis. Quantitative evaluation of the BBB damage was performed using the Evans blue (EB) extravasation assay. As shown in Figure 5C, nicotine was able to more significantly increase E44-induced permeability of the BBB in wildtype mice (P<0.01) when compared to that in KO animals (P<0.05). The enhanced permeability of the BBB induced by E44 cells was significantly increased in wildtype mice as compared to KO mice. These results suggest that α7 nAChR is required for pathogen- and nicotine-increased BBB permeability. As shown in images of mouse brains with EB staining (Figure 5D), substantially heavier EB staining was seen in wildtype animals treated with nicotine than in other treatment settings. Alternatively, the permeability of the BBB was examined by measuring albumin in CSF samples, as described previously [33]. Nicotine-enhanced albumin passage across the BBB was reduced by either chemical (MLA) (Figure S2C) or genetic (KO) (Figure S2D) blockage of α7 nAChR. Accordingly, albumin passages across the BBB were also increased in nicotine-treated heterozygous mice (Figure S3E) and the WT mice with tobacco smoking (Figure S4E). These results confirmed the conclusion that α7 nAChR contributed to modulation of the BBB permeability.

### Effects of α7 deficiency on *E. coli* K1- and nicotine-induced impairment of tight junction

**Figure 6:**
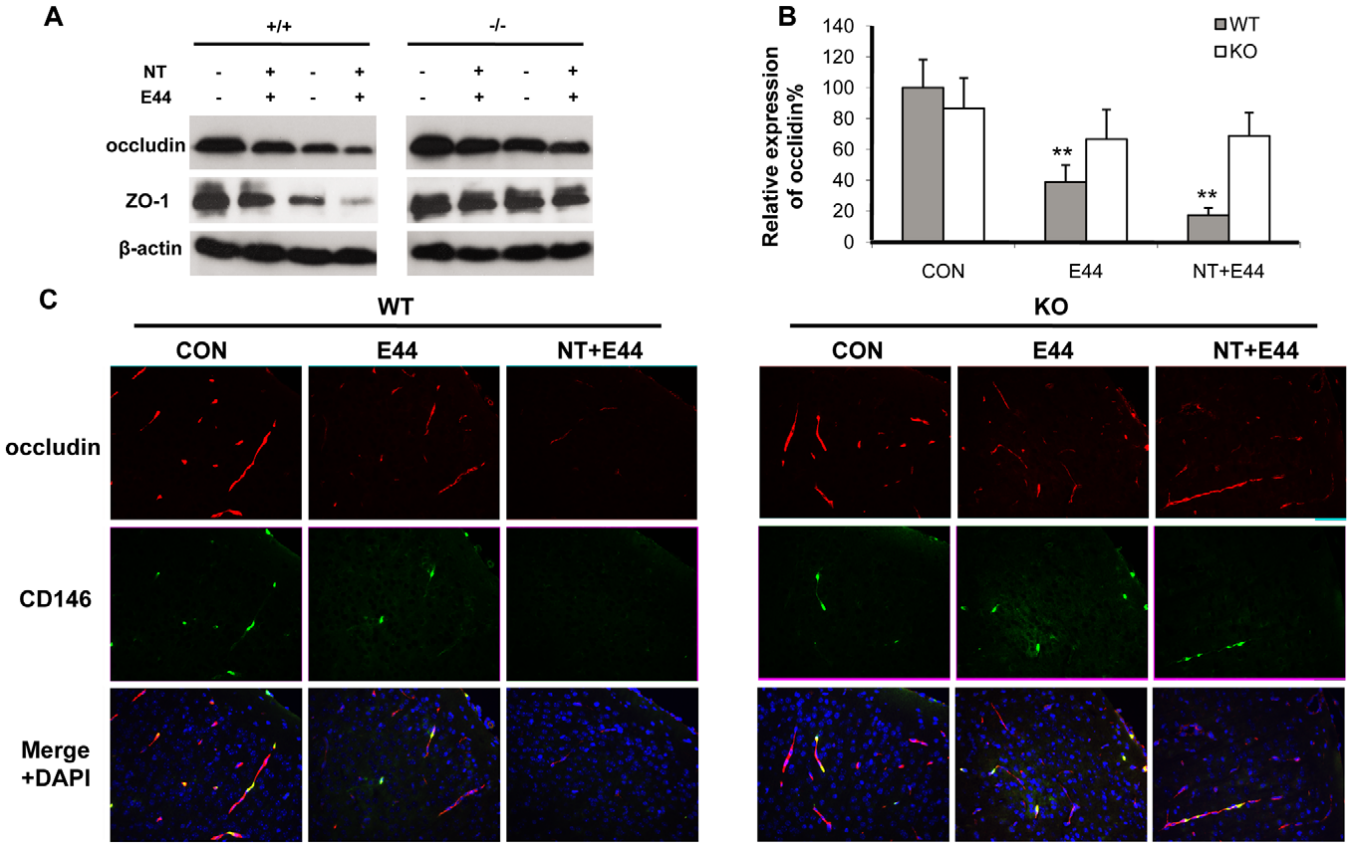
Effects of α7 knockout on nicotine- and E44-induced disruption of tight junction (TJ). (**A**) Immunoblotting showed the expression of tight junction molecules occludin and ZO-1 in WT and KO MBMEC upon treatment with nicotine (10 µM for 48 h) and E44 (10^6^/ml for 4h) alone or in combination. β-actin was used as an internal loading control. (**B**) Quantification of occludin expression in WT and KO mouse (n = 5-6) brain cortex upon treatment with or without NT and E44. The fluorescence intensities of occludin were quantified and expressed as relative expression compared to the controls. The control (WT) without any treatment was taken as 100%. (**C**) Immunostaining of TJ molecules in mouse brain cortex with or without NT exposure and E44 infection using antibodies against occludin (rhodamine-conjugated). A FITC-conjugated anti-CD146 Ab was used to stain MBMEC, and DAPI was used to stain the structures of brain cortex in the merged pictures. CON: mice without any treatment. E44: mice infected with E44. NT+E44: NT-treated mice infected with E44. Images are 200×.

To compare the integrity of the BBB *in vitro* and *in vivo* upon stimulation with nicotine and E44, the tight junction molecules occludin and ZO-1 were examined by immunoblotting and immunohistochemical staining. Immunoblotting indicated that nicotine could decrease the expressions of ZO-1 and occludin in a dose- (0.1–10 µM) and time-dependent (0–72 h) manner in WT MBMEC, while expression of α7 nAChR was increased in a dose- and time-dependent fashion during the treatments (Figure S5A and S5B). It concurred with a previous report that nicotine could upregulate α7 nAChR through activation of nuclear transcription factor kappa B [34]. Chemical blockage of α7 nAChR by MLA could reverse the effects of nicotine on ZO-1 and occludin expressions in a dose-dependent manner, while the up-regulated expressions of α7 nAChR by nicotine were reduced to the basal level (Figure S5C). Then, WT and KO MBMEC were treated with nicotine and E44 alone or in a combination. A greater decrease in expression of ZO-1 and occludin was observed in the combination settings than either treatment alone in WT MBMEC (Figure 6A). However, these effects were significantly reduced in KO MBMEC, suggesting that the α7 deficiency could protect the tight junction from nicotine- and bacteria-induced degradation. Immunohistochemical staining of occludin and ZO-1 in mouse brain cortex were consistent with the *in vitro* data. As shown in Figure 6C, E44 infection significantly reduced occludin expression in the cortex. A combined treatment with nicotine and E44 resulted in an additive or synergistic effect of decreased occludin expression in the brain tissues, which was much lower than that in other treatment settings in the wildtype mice. These results showed that both *E. coli* K1 and nicotine could induce BBB damage by decreasing expression of tight junction molecules. However, E44 and nicotine only induced slight changes in occludin expression in the brains of KO mice, suggesting that α7 nAChR is required for E44- and nicotine-induced BBB damages. The quantification analysis of occludin fluorescence intensity was showed in Figure 6B, confirming the detrimental role of α7 nAChR to BBB. Similar results were obtained when examining ZO-1 expression (Figure S5D and S5E). These results suggest that α7 nAChR contributes to pathogen- and nicotine-increased BBB permeability by decreasing protein levels of tight junction molecules.

### Neuronal injury in the hippocampus is reduced in α7^-/-^ mice with *E. coli* meningitis

**Figure 7:**
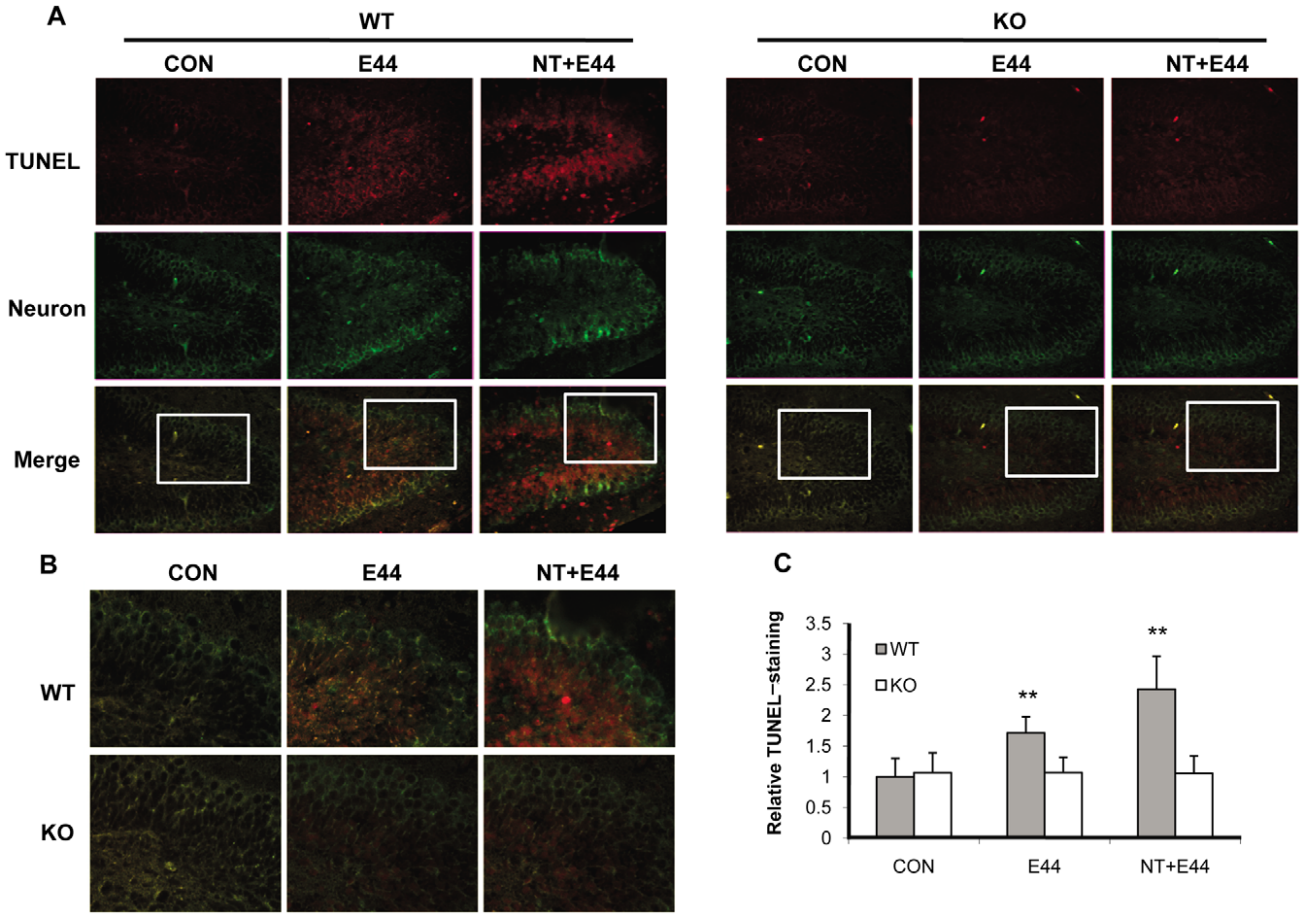
Reduced neuronal injury in the hippocampus of α7 KO mice. (**A**) Neuron damage in the dentate gyrus of hippocampus within WT and KO mouse brains was examined by the TUNEL assay. Neurons were stained with an antibody against neuron nuclear protein (NeuN) (FITC-conjugated). Apoptotic cells were stained with rhodamine after the TUNEL assay. CON: mice without any treatment. E44: mice infected with *E. coli* K1. NT+E44: NT-treated mice infected with E44. Images are 100×. The squared areas in merged pictures were enlarged in (**B**) to show the details of TUNEL staining. Images are 200×. (**C**) Quantification of TUNEL-staining in the dentate gyrus of hippocampus within WT and KO mice (n = 5-6) treated with or without NT and E44. The fluorescence intensity of TUNEL staining was quantified with the dentate gyrus of hippocampus and expressed as relative expression compared to the controls. The control (WT) without any treatment was taken as one fold.

Bacterial meningitis causes neuronal damage that predominates in the hippocampal dentate gyrus [35]. In light of this, we next examined the neuronal injury in the hippocampus in the murine model of *E. coli* meningitis using the TUNEL assay for detecting apoptotic neurons and co-staining with an antibody against mature neurons. As shown in Figure 7A and 7B, no or few TUNEL-positive neurons were found in the dentate gyrus of the hippocampus within untreated wildtype and KO mouse brains. *E. coli* infection significantly induced TUNEL-positivity in neurons of the inner layers of dentate gyrus in wildtype mouse brains, but not in KO mouse brains. Nicotine dramatically enhanced *E. coli* virulence as measured by the induction of neuronal apoptosis in wildtype mouse brains; however, only a few apoptotic neurons were found in KO mouse brains as compared to the control. The quantification analysis of TUNEL staining fluorescence intensity was shown in Figure 7C, confirming the detrimental role of α7 nAChR in neuronal injury. These data demonstrated that the deficiency of α7 nAChR was neuroprotective for neonatal mice with *E. coli* meningitis.

### Cerebrospinal fluid (CSF) cytokine levels are reduced by chemical (MLA) and genetic (α7 KO) blockage of α7 nAChR during *E. coli* K1 meningitis

**Figure 8:**
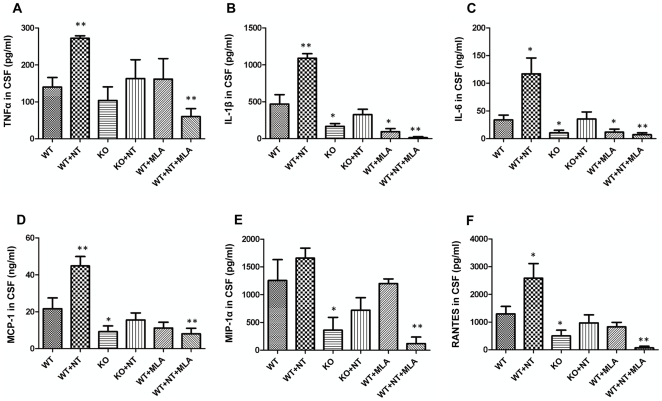
Decreased CSF cytokine levels in α7 KO mice. Inflammatory cytokine levels in mouse CSF after *E. coli* infection were examined by the Cytometric Beads Array (CBA) as described in Methods and Materials. The values were expressed as concentrations of cytokines (pg/ml or ng/ml) representing means of 4-5 samples for each group. WT mice served as the control (*P<0.05; **P<0.01).

To further determine the role of α7 nAChR in the CNS inflammation, the levels of cytokines in CSF samples were measured using the Cytometric Beads Array (CBA) assay as described in Methods and Materials. This technique is able to quantify multiple proteins simultaneously with the use of the broad dynamic range of fluorescence detection offered by flow cytometry and antibody-coated beads to efficiently capture analytes. The levels of cytokines in CSF, including IL-1β, IL-6, TNFα, MCP-1, MIP-1α and RANTES were analyzed (Figure 8A-F). The data showed that nicotine could significantly increase the levels of all of these cytokines except MIP-1α, while the deficiency of α7 nAChR resulted in a significant decrease of these cytokines. Nicotine was unable to significantly upregulate these cytokines in KO mice. Meningitic *Cryptococcus neoformans* was unable to up-regulate expression of cytokines under the similar experimental settings (data not shown), suggesting that induction of pro-inflammatory factors is pathogen-dependent. In addition, the α7 antagonist MLA could inhibit these cytokines in nicotine-treated mice. These data suggested that α7 nAChR could upregulate inflammatory cytokines in *E. coli* K1 meningitis.

### *E. coli* K1- and nicotine-increased protein levels of adhesion molecules ICAM-1 and CD44 were reduced in α7-deficient cells and animals

**Figure 9:**
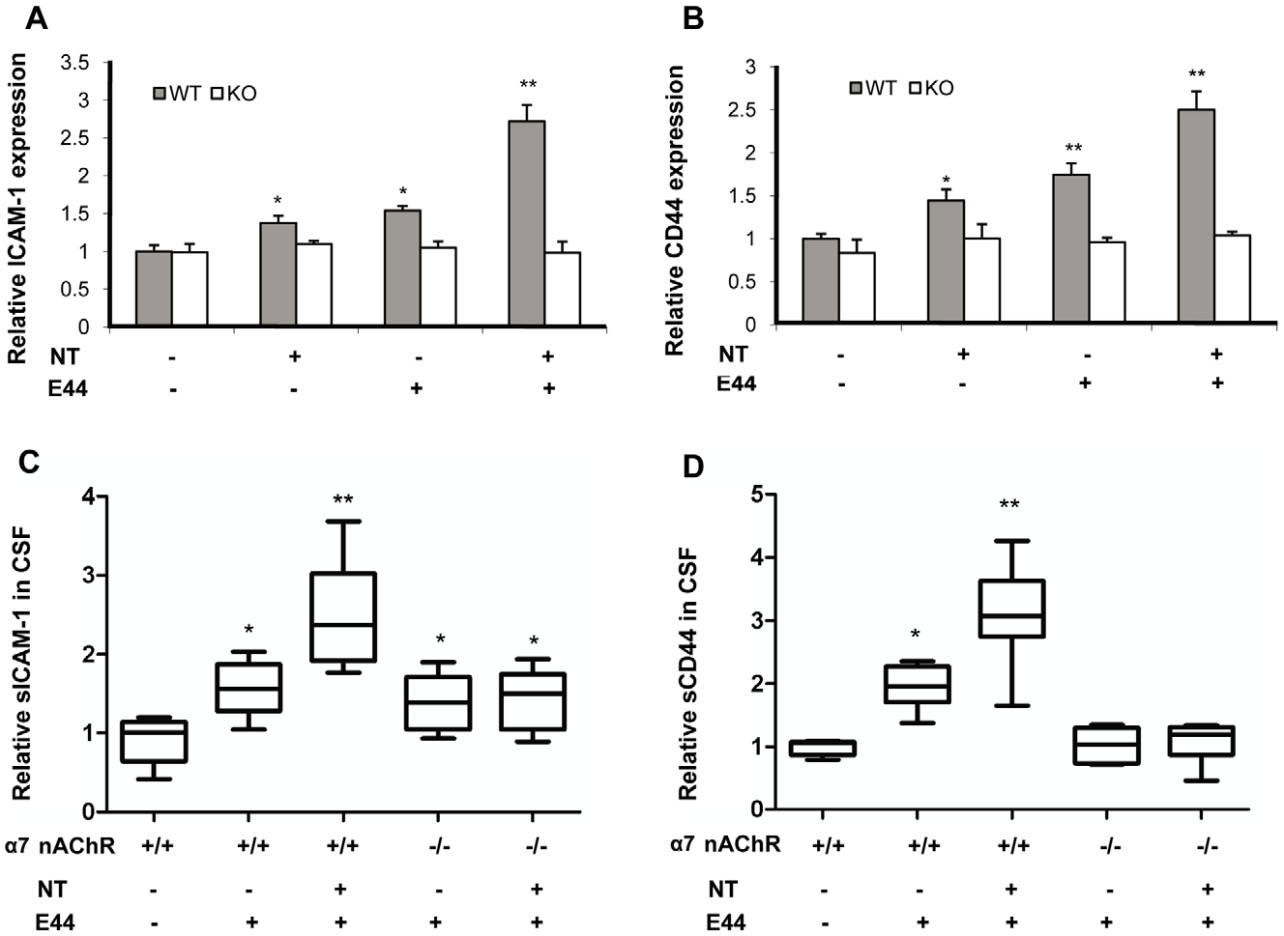
Decreased expression of ICAM-1 and CD44 in α7^-/-^ MBMEC and mice. (A) and (B) Expression analysis of cell surface ICAM-1 and CD44 in α7^-/-^ MBMEC upon treatment with NT and *E. coli* K1. WT and KO MEBMC were cultured in 96-well plates, exposed to NT (10 µM) for 48 hours, and incubated with E44 cells (10^6^/ml) for 4 hours. Cells were fixed and subjected to ELISA for ICAM-1 and CD44 as described in Methods and Materials. WT MBMEC without any treatment was taken as a control and set as one fold. (C) and (D) Decreased levels of soluble ICAM-1 and CD44 in KO mouse CSF. WT and KO mouse CSF samples were subjected to ELISA assays for the expression of ICAM-1 and CD44 as described in Methods and Materials. Values were expressed as relative expression. WT mice without any treatment severed as controls, and their means were defined as one-fold. (*P<0.05; **P<0.01).

As our previous study has shown that *E. coli* K1-increased expression of adhesion molecules ICAM-1(CD54) and CD44(HCAM) in HBMEC is required for PMN binding and transmigration [27], we examined their expression in α7^-/-^ cells and mice. As shown in Figure 9A and B, both nicotine and E44 could increase the expression of ICAM-1 and CD44 in α7^+/+^ MBMEC, and the expression was dramatically up-regulated with combination of nicotine and E44. However, there were no significant changes in expressions of ICAM-1 and CD44 in α7^-/-^ MBMEC, suggesting that α7 nAChR is required for both nicotine- and *E. coli* K1- induced expression of the analyzed adhesion molecules. To validate the relevance of the *in vitro* results, CSF samples taken from neonatal mice with meningitis (positive bacterial cultures in brain tissues) were used to examine the levels of soluble ICAM-1 and CD44. Results were consistent with the *in vitro* findings, which showed that E44 infection could increase the expression of ICAM-1 and CD44 in wildtype mice, and that nicotine could amplify E44-induced expression of these two adhesion molecules in the wildtype animals (Figure 9C and D). However, there is little difference in their expression levels in KO mice treated with either E44 or a combination of E44 and nicotine. In addition, accumulation of soluble CD44 in the CSF was significantly reduced in KO mice compared with wildtype animals after *E. coli* infection, suggesting that α7 nAChR contributes to up-regulation of adhesion molecules induced by both nicotine and *E. coli* K1.

### α7 nAChR-mediated calcium signaling contributed to *E. coli* K1-induced bacterial invasion and PMN transmigration

**Figure 10:**
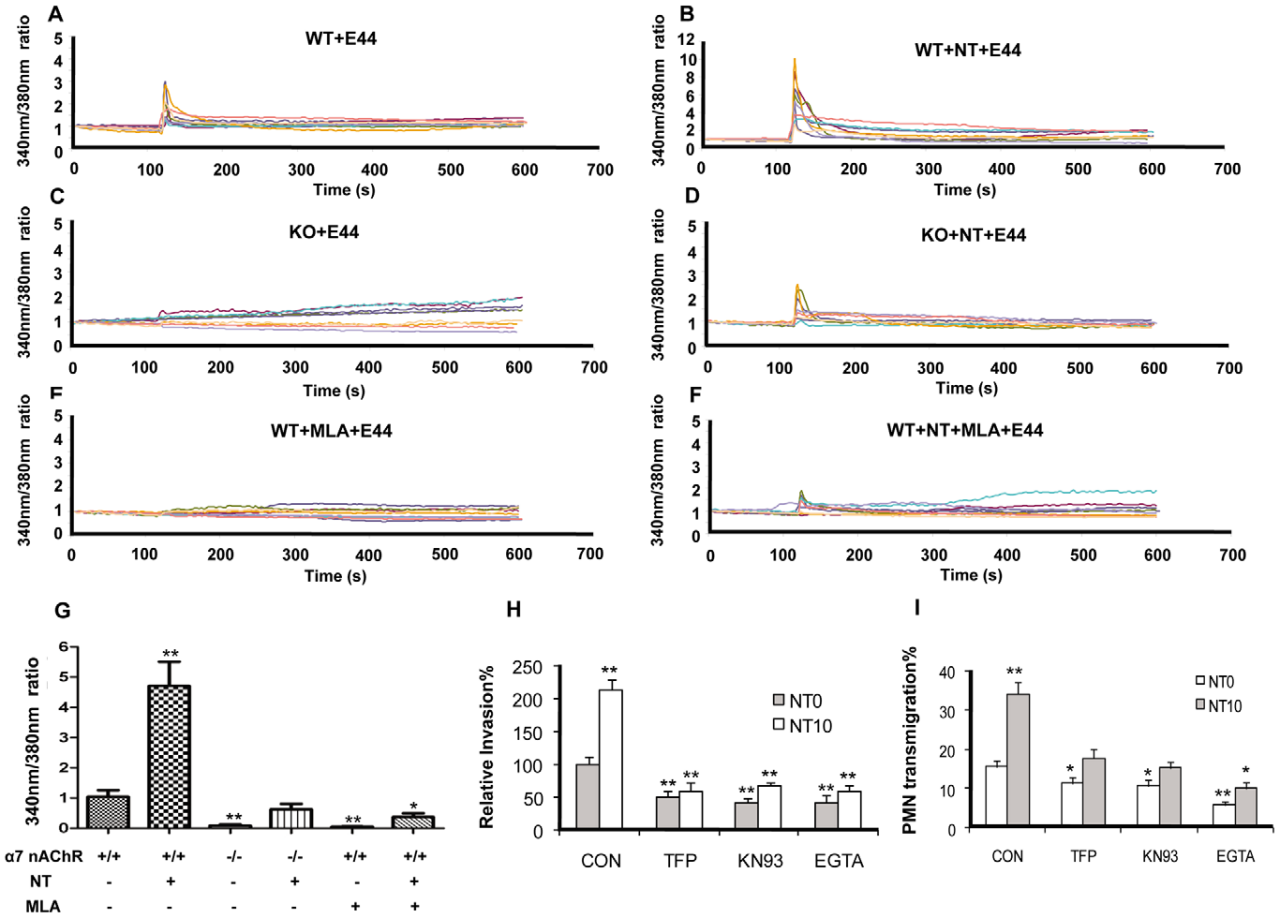
Involvement of α7 nAChR in *E. coli* K1-induced intracellular calcium flux. (**A–F**) Elevation of intracellular calcium flux in MBMEC stimulated with E44 cells. WT and KO MBMEC monolayers were exposed to NT (10 µM) or MLA (1 µM) for 48 hours, and then loaded with Fura-2 AM as described in Methods and Material. The monolayer was monitored for intracellular calcium flux for 10 minutes with 4 s intervals under an automated fluorescent microscope. Monolayer cells were stimulated with E44 cells (10^8^ CFU) at the 120 s time point. The intensity of fluorescence at 340 nm and 380 nm was measured. The ratios of intensity of fluorescence at 340 nm and 380 nm were calculated for each time interval and depicted as continuous lines in (**A–F**). The y axis represents the ratio, and x axis represents time (s). For each treatment, measurements were repeated with nine replicates and represented with different colors. (**G**) The 340 nm/380 nm ratio changes in each treatment were calculated and subjected to statistical analysis. WT MBMEC without any pre-treatment served as a control and defined as one-fold (1.0). (**H**) and (**I**) NT-enhanced E44 invasion and PMN transmigration in WT MBMEC was blocked by inhibitors of calcium signaling, TFP, KN93, and EGTA. MBMEC were pre-incubated with or without NT (10 µM) for 48 hours, and then treated with TFP (5 µM), KN93 (25 µM) and EGTA (100 µM) for 1 hour before the invasion or PMN transmigration assays. For the invasion assays, results are expressed as relative invasion compared to the positive control without any treatment (100%). All invasion assays were performed in triplicate wells. For the PMN transmigration assays, values represent the means of transmigrating PMN (%) in triplicate samples. Bar graphs showed the means ± SD of triplicate samples. In both invasion and PMN transmigration assays, significant differences with regard to the controls (MBMEC without any treatment) were marked by asterisks (*P<0.05; **P<0.01).

Calcium signaling has been found to be important for the pathogenesis of bacterial infection [20] and the biological functions of α7 nAChR [18]. The *E. coli* K1 virulence factor is able to increase intracellular transient calcium flux in human BMEC [22], but the underlying mechanism is unknown. Based on the above findings and the relatively high calcium permeability of the α7 nAChR ion channel, we hypothesized that α7 nAChR-mediated calcium signaling might be the major regulatory pathway for the CNS inflammatory response to bacteria and other pathogenic insults, including nicotine. To test this hypothesis, we examined the role of α7 nAChR in E44- and nicotine-induced signaling using wildtype and KO MBMEC. Fura-2 AM, a calcium fluorescence dye, was used for measurement of intracellular free calcium. Changes in the ratio of 340 nm/380 nm were calculated as representing the strength of calcium flux. The ratio changes occurred immediately in wildtype MBMEC upon E44 stimulation with a range of 0.5-3 fold increase (Figure 10A). Much higher ratio changes (3-10 fold) were observed in the same cells stimulated with E44 after exposure to nicotine for 48 hours (Figure 10B). These results indicated that nicotine could amplify the transient intracellular calcium flux induced by E44, which might be the initial step of bacterial invasion. However, KO MBMEC did not exhibit significant ratio changes upon stimulation with E44 (Figure 10C), suggesting that α7 nAChR might be the major pathway for the *E. coli* K1-induced calcium flux. KO MBMEC exhibited much lower fold increase in ratio changes than in wildtype cells under the same treatment settings (E44 plus nicotine) (Figure 10D). These results showed that the deficiency of α7 nAChR significantly reduced the intracellular calcium flux upon stimulation with nicotine and E44. However, KO MBMEC showed a slight increase in the ratio upon co-stimulation with nicotine and E44 as compared to the same cells without nicotine treatment, suggesting that there might be non-α7 nAChRs that interact with nicotine. To further confirm the role of α7 nAChR in nicotine- and E44-induced calcium signaling, MLA-mediated chemical blocking was also tested. The result showed that MLA could completely abolish the *E. coli* K1-induced calcium flux in wildtype MBMEC without nicotine treatment (Figure 10E), and significantly reduce the ratio changes in wildtype MBMEC exposed to nicotine (0-2 fold, Figure 10F), suggesting that *E. coli* K1-induced calcium flux was entirely dependent on α7 nAChR. Statistical analysis indicated that either chemical (MLA) or genetic (KO) blockage of α7 nAChR could significantly inhibit E44-induced intracellular calcium flux in MBMEC with or without nicotine exposure (Figure 10G). These results were consisted with the conclusion drawn from the studies with KO MBMEC. To further confirm this conclusion, inhibitors of the calcium signaling pathway, including inhibitors of calmodulin [trifluoperazine (TFP)] and calmodulin kinase II (KN93), and the calcium chelating agent EGTA, were tested for their ability to block bacterial invasion and PMN transmigration. As shown in Figure 10H and 10I, these inhibitors could significantly block *E. coli* K1 invasion and PMN transmigration in wildtype MBMEC with or without nicotine exposure. These data suggest that α7 nAChR-mediated calcium signaling contributes to nicotine-mediated stimulation, *E. coli* K1 invasion and PMN transmigration across the BBB.

## Discussion

Currently, the mechanisms responsible for the modulation of the host response to microbial infection are incompletely understood, but overwhelming evidence suggests that there are active connections between the nervous, endocrine, and immune systems during the regulation of inflammatory processes in various types of cells and tissues [4], [36]. The cholinergic α7 nAChR pathway has recently been found to play an essential role in regulation of host inflammatory response to microbial infection [3]-[4], [8]. Since the activation of the α7 receptor, the major subtype of neuronal nAChRs, has deleterious effects on neonatal brain injuries [15], an understanding of the early inflammatory response to meningitic infection is important for the prevention and treatment of neonatal bacterial meningitis. We were interested, therefore, in dissecting the regulatory role of α7 nAChR in the host defense against meningitic infection. In this report, we have established that α7 nAChR plays a detrimental role in host defense against bacterial meningitis in the mouse model. The entrance of pathogens and leukocytes into the CNS, which is correlated with increased BBB permeability, is significantly reduced in the α7-deficient mice. Calcium signaling mediated by α7 nAChR is the major regulatory pathway for the CNS inflammatory response to meningitic *E. coli* infection and nicotine exposure. The resulting neuronal inflammation, including secretion of proinflammatory factors (IL-1β, IL-6, TNFα, MCP-1, MIP-1α, RANTES, CD44 and ICAM-1) into the CSF and inflammatory response in the hippocampus, is significantly reduced in α7-deficient mice during *E. coli* meningitis. Furthermore, these findings are consistent with clinical observations in humans of an increased incidence of bacterial meningitis as a consequence of exposure to second hand tobacco smoke containing nicotine, an α7 agonist that enhances α7 nAChR activation. These findings provide insight into an element of host defense previously unknown to influence the susceptibility to bacterial meningitis, and present novel opportunities to improve disease outcome in humans.

The cholinergic α7 nAChR pathway-mediated inflammatory regulation has been extensively investigated in models of experimental sepsis, endotoxemia, ischemia/reperfusion injury, hemorrhagic shock, arthritis, and other sterile inflammatory disorders [37]. However, studies on its role in the innate immune response to microbial infection are very limited. These include a few *in vitro* studies on bacterial infection involving chemical stimulation and blockage of the cholinergic pathways [8], [38], and a recent investigation on bacterial peritonitis by genetic blockage of α7 nAChR [4]. In order to determine whether and how α7 nAChR plays a role in the pathogenesis of bacterial meningitis, we first established the *in vitro* and *in vivo* mouse models of the BBB with a combination of endogenous/exogenous and chemical/genetic approaches for inhibition and stimulation of the cholinergic α7 nAChR pathway. The combined approaches could maximize their advantages and minimize their disadvantages. Both *E. coli* K1 invasion and PMN transmigration were significantly reduced in α7^-/-^ MBMEC and α7^-/-^ mice when compared to that in the wildtype cells and animals. The α7 KO cells and mice were unable to generate a response to α7 agonist (nicotine)-mediated stimulation during bacterial infection. The α7 antagonist MLA was able to block nicotine-mediated stimulation in WT mice upon infection with *E. coli* K1. These findings suggest that α7 nAChR plays an essential role in regulation of the host inflammatory response to meningitic *E. coli* K1 infection. Thus, the present report is the first to use α7^-/-^ cells and mice to dissect the role of the cholinergic α7 nAChR pathway in host defense against meningitic infection.

The most critical step in the pathogenesis of bacterial meningitis is the penetration of the extracellular pathogens across the BBB, a formidable defense system that normally keeps out pathogens and toxins. It has been demonstrated that nicotine is able to modulate the BBB permeability through the cholinergic α7 nAChR pathway [39]. We and others have demonstrated that multiple bacterial virulence factors, including Ibe proteins (IbeA, IbeB, IbeC and IbeT), AslA (arylsulfatase-like gene), FimH (type 1 fimbrial tip adhesin), TraJ (positive regulator of the F plasmid transfer (tra) operon) and OmpA (outer membrane protein A) are able to breach the BBB [27]. The precise mechanism responsible for the *E. coli* K1-mediated increase in BBB permeability during meningitis is largely unknown. Although it is well-known that proinflammatory factors promote increased BBB permeability, it is unclear how the production of these factors is regulated during this disease. In this investigation, our results show that α7 nAChR is able to directly or indirectly upregulate proinflammatory factors and has a detrimental effect on the permeability of the BBB in the early stages of meningitic infection. It is most likely that the α7 receptor-upregulated production of proinflammatory factors results in increased BBB permeability, which facilitates the entrance of pathogens and leukocytes into the CNS. This notion is further supported by our finding that the α7 KO mice with direct inoculation of *E. coli* K1 into the CSF show reduced bacteremia and CNS inflammatory response (*e.g*., decreased PMN recruitment and albumin leakage into CSF) when compared to that in the wildtype animals (data not shown). This suggests that accelerated bacterial clearance in α7 KO mice occurs.

The observation that α7-deficient BMEC were unable to increase intracellular calcium concentrations upon stimulation with either pathogens or the α7 agonist nicotine provides two key pieces of information that are critical for elucidating the molecular mechanism behind the α7 receptor-mediated suppression of the host defense against bacterial meningitis. First, Ca^2+^ signaling has been implicated in meningitic *E. coli* K1 infection [22]. FimH, which is regulated by IbeA [40], can induce an increase in free cytosolic calcium in human BMEC. Phosphorylation of the IbeA receptor vimentin by Ca^2+^/CaMKII and activation of ERK1/2 are required for IbeA+ *E. coli* K1 invasion of human BMEC [23]. The current investigation demonstrated that nicotine and *E. coli* K1 could additively or synergistically increase intracellular Ca^2+^ concentrations through the cholinergic α7 nAChR pathway. Pathogen-induced calcium fluxes in MBMEC were almost completely abolished by either chemical (α7 antagonist MLA) or genetic (KO cells) blockage. On the other hand, the nAChRs are a family of ligand-gated calcium channels formed by a pentameric complex of nAChR subunits [3]. Since the α7 receptor is the major subtype of nAChRs in the CNS, it plays an important role in calcium signaling in neuronal and non-neuronal cells through regulation of intracellular calcium, which leads to activation of signal transduction pathways, including ERK1/2, CREB, and AKT [17]. Nicotine is able to activate the Ca^2+^/calmodulin signaling pathway through the α7 receptor [18]. Ca^2+^/CaMKII can be activated by galantamine, a novel Alzheimer's drug, which is known to inhibit acetylcholinesterase activity and potentiate nicotinic acetylcholine receptor (nAChR) in the brain [41]. Our results demonstrate that KN93, a specific inhibitor of Ca^2+^/CaMKII, is able to block nicotine-enhanced *E. coli* K1 penetration across BMEC [23]. These findings suggest that the Ca^2+^/calmodulin signaling pathway is commonly activated upon meningitic infection with pathogens and stimulation of the α7 receptor. Thus, meningitic pathogens and nicotine can additively or synergistically induce the cellular release of Ca^2+^ that may expand bacterial cell signaling through the cholinergic α7 nAChR pathway, leading to enhanced bacterial invasion and leukocyte transmigration that are associated with the BBB disorders, and increased host susceptibility to the invading microorganism. However, the underlying molecular mechanisms that activate α7 nAChR-mediated calcium signaling and the exact nature of where these signaling molecules are assembled and regulated remain elusive. Research from several groups has demonstrated that lipid rafts/caveolae can serve as microdomains of calcium signaling through clustering of Ca^2+^ channels and their regulators in such platforms [42]. We have recently shown that *E. coli* K1 and nicotine could increase the recruitment of α7 nAChR and related signaling molecules, including vimentin, and Erk1/2, to caveolin-1 enriched lipid rafts [24]. Synergistic effects were observed upon treatment with a combination of *E. coli* K1 and nicotine. These findings suggest that lipid rafts/caveolae could provide a favorable platform for cross-talk between the cholinergic signaling pathway (*e.g.*, α7 nAChR/CaMKII/ERK1/2) and non-cholinergic signaling pathways (*e.g.*, vimentin/CaMKII/ERK1/2).

It is worth noting that there may be a difference between neonatal and non-neonatal patients regarding the role of α7 nAChR in neuronal injury during bacterial meningitis. It has been reported that activation or suppression of α7 nAChR in the CNS has opposite effects on neonatal excitotoxic brain injuries when compared to that in adults [15]. Activation of α7 is protective in adult animals but deleterious in neonatal mice, whereas its blockade, either pharmacologically (α7 antagonist) or genetically (α7^-/-^ mice), provides neuroprotection. However, it has been shown that there is no difference between neonates and adults in the deformability and volumes of leukocytes, which are essential for PMNs emigration from the intravascular to the extravascular space [43]. This suggests that α7 nAChR may differentially contribute to modulation of the host inflammatory responses in different tissues to different disease conditions. In the sterile inflammatory disorder model used by Wang *et*. *al*. [3], α7 nAChR plays an anti-inflammatory role in the host response against endotoxin. However, this receptor contributes oppositely to the host response to bacterial infections, including *E. coli* peritonitis [4] and *E. coli* meningitis (this report). *E. coli* meningitis commonly occurs in the neonatal period [1], [2], but the basis of this age dependency is largely unclear. The α7 nAChR cholinergic pathway may be differentially regulated in an age-dependent manner. Although α7 nAChR plays a major role in the cholinergic anti-inflammatory pathway, the other major subtype of nAChRs in the CNS, α4β2, partially mediate the anti-inflammatory response, which is not dependent on calcium signaling [44], suggesting that multi-subtypes of nAChRs may contribute to the cholinergic regulation of inflammatory response in an elegant manner. Nicotine, which can interact with the two major nAChRs (α7 and α4β2) in the CNS, is able to significantly increase the levels of proinflammatory factors (IL-1β, IL-6, TNFα, MCP-1, MIP-1α, RANTES) in CSF. The wildtype mice treated with nicotine and MLA (α7 antagonist) had decreased responses relative to the animals left untreated or treated with MLA alone, suggesting that multi-subtypes of nAChRs may contribute to the cholinergic regulation of proinflammatory responses. MLA may be capable of antagonizing endogenous α7 agonists such as acetylcholine and choline or competing with endogenous α7 inhibitors such as catestatin [36]. Considering the possible involvement of multi-subtypes of nAChRs in meningitic infection and the opposite effects of α7 nAChR activation/suppression on neonatal excitotoxic brain injuries in neonates and adults, close attention must be paid to the pathogenesis and therapeutic manipulations of neonatal and non-neonatal bacterial meningitis.

Collectively, the major finding of the present report is that α7 nAChR deficiency is protective against meningitic infection by down-regulation of pathogen invasion, PMN recruitment, calcium signaling and neuronal inflammation. Further insight into how meningitic pathogens utilize the host cholinergic α7 nAChR pathway to augment their virulence capacity will advance our understanding of the pathogenesis and therapeutics of bacterial meningitis.

## Methods and Materials

### Ethics statement

This study was performed in strict accordance with the recommendations in the Guide for the Care and Use of Laboratory Animals of the National Institutes of Health. Our protocols were approved by the Institutional Animal Care and Use Committee (IACUC) of The Saban Research Institute of Children's Hospital Los Angeles (Permit number: A3276-01). All surgery was performed under anesthesia with ketamine and lidocaine, and all efforts were made to minimize suffering.

#### Chemicals and reagent

Dextran, Evans blue, nicotine tartrate (NT), MLA, TFP, and ethylene glycol tetraacetic acid **(**EGTA) were purchased from Sigma-Aldrich (St. Louis, MO). Dynabeads M-450 Tosylactivated, α–bungarotoxin (α–BTX) tetramethylrhodamine conjugate, Fura-2 AM, Pluronic-127 were purchased from Invitrogen (Carlsbad, CA). Ulex europaeus I (UEA I) lectin and mounting medium with DAPI were purchased from Vector (Buringame, CA). KN93 was purchased from ALEXIS Biochemicals (San Diego, CA). All primary antibodies (Ab) were purchased from the commercial sources: a rabbit anti-ZO-1 Ab (33-1500) and a mouse anti-occludin Ab (61-7300) from Invitrogen; a rabbit anti-α7 nAChR Ab from Genescript (Piscataway, NJ); a rat anti-mouse Ly-6G (Gr-1) Ab, a mouse anti-neuron (NeuN) Ab from eBiosciences (San Diego, CA); a mouse anti-CD44 Ab (sc-7297), a rabbit anti-β-actin (sc-7210), and a rabbit anti-GGT Ab (sc-20638) from Santa Cruz Biotechnology (Santa Cruz, CA); an anti-mouse CD146 Ab FITC-conjugated from Biolegend (San Diego, CA), a rabbit anti-S100B Ab from BD Biosciences, and a rabbit anti-CD54 Ab (ICAM-1, 250593) from Abbiotec (San Diego, CA). The TUNEL assay kit was purchased from Millipore (Chemicon, Billerica, MA). Transwell filters (3 µm pore size, 6.5 mm diameter), blood plates and CBA assay kit were purchased from BD Biosciences (San Jose, CA).

#### Mice

Heterozygous (+/−) α7-deficient mice with the C57BL/6J background (B6.129S7-Chrna7^tm1Bay^/J) were purchased from Jackson Laboratory (Bar Harbor, ME). Genotypes of α7 ^+/+^ mice (WT mice), α7^-/-^ mice (KO mice) and heterozygous α7 ^+/-^ mice were determined according to the PCR protocol provided by the vendor. The animals were used in transgenic breeding at 8 weeks of age for optimum reproductive performance. Male heterozygous (+/−) and female homozygous (-/-) were used in breeding. The average litter size for neonatal mice was 6–8. Age- and sex-matched mice were used in all experiments. All experiments were approved by the Animal Care and Use Committee of Childrens Hospital Los Angeles Saban Research Institute.

### Isolation and purification of mouse brain microvascular endothelial cells

Isolation of mouse BMEC was performed with Ulex europaeus I (UEA I) lectin-coated Dynabeads as described previously [45]. The beads were prepared according to the manufacturer's instructions (Invitrogen) and resuspended in Hanks' balanced salt solution (HBSS, Invitrogen Corp., Carlsbad, CA, USA) plus 5% fetal calf serum (HBSS+5 %FCS) to a final concentration of 4×l0^8^ beads/ml. The MBMEC were prepared as described previously [46]–[47]. Briefly, the mouse (10-day-old) brain specimens devoid of large blood vessels were homogenized in HBSS and centrifuged in 12.5 % dextran (Mr∼70,000, Sigma) at 8,000 g for 10 min. Pellets containing crude microvessels were further digested in a solution containing collagenase (0.1 U/ml), dispase (0.8 U/ml) and DNase I (10 U/ml, Sigma). Microvascular capillaries were isolated by absorption to Ulex-coated beads. The confluent MBMEC monolayer displays a cobblestone appearance when grown on collagen-coated surfaces. The cells were positive for CD146 [48], demonstrating their endothelial origin, and also expressed S100B [49] and GGT [46], indicating their brain origin. MBMEC exhibited an average TEER value of 250–300 Ω/cm^2^ [50]. The cells also exhibited the typical characteristics for brain endothelial cells expressing tight junctions and a polarized transport of rhodamine 123, a ligand for P-glycoprotein [51].

#### *E. coli* strain and invasion assay

E44, a rifampin-resistant derivative of *E. coli* K1 strain RS218 (serotype 018:K1: H7) [1], [52], was grown for 15 h at 37°C in L broth in the presence of rifampin (100 µg/ml). To test the effects of nicotine on *E. coli* invasion, MBMEC were subcultured in tissue culture plates and 1×10^-5^ to 10^−7^ M of nicotine tartrate was pre-incubated with MBMEC in RPMI-1640 medium. After exposure to nicotine, cell cultures were examined under a microscope. *E. coli* invasion assays were performed as described previously [1], [8]. The released intracellular bacteria were enumerated by plating on sheep blood agar plates. Cell viability was routinely verified by the trypan blue staining assay. Results were expressed as relative invasion (percentage of invasion in comparison to that of untreated MBMEC). The α7 antagonist MLA, Ca^2+^ pathway inhibitors KN93, TFP, and EGTA were used to examine the role of α7 in nicotine-enhanced *E. coli* invasion. The inhibitors were incubated with the MBMEC monolayers for 1h at 37°C before addition of bacteria. All inhibitors were present throughout the invasion experiments until the medium was replaced with experimental medium (EM) containing gentamicin. The effect of these inhibitors on *E. coli* and MBMEC was examined by bacterial colony counting and trypan blue staining methods, respectively.

#### PMN transmigration

Mouse PMNs were isolated according to standard techniques from heparin anticoagulated venous blood of 8-10 week-old mice for both α7 nAChR wildtype and KO mice [27]. The isolated mouse PMN were 99% pure as indicated by immunostaining with an antibody against the Ly-6G neutrophil marker. Leukocyte transmigration assays were performed as described previously [27], [53]–[54] with modification. To test the effects of nicotine on PMN transmigration, MBMEC were subcultured on transwell filters (3.0-µm pore size, 6.5mm diameter) and exposed to nicotine as described above. The confluence of the monolayer was confirmed by light microscopy before the start of the assay. E44 (10^5^ CFU/ml) was added to the lower chambers and incubated for 2 h. Then, PMN (1×10^6^ cells) were added to the upper chamber and allowed to migrate over for 4 h. At the end of the incubation, migrated PMN cells were collected from the lower chamber and counted as described previously [27]. All experiments were performed with triplicate wells. For inhibitions of PMN transmigration, cells were incubated with inhibitors for 1 h before E44 stimulation. All inhibitors were present throughout the experiment. The BMEC monolayers on Transwell filters were monitored before and after PMN migration by measuring trans-endothelial electrical resistance (TEER) changes in the endothelial cell monolayer using a Millipore ERS apparatus, according to manufacturer's instruction.

#### PMN binding

PMN adhesion assays were performed as described previously [27], [55]. Briefly, mouse BMEC monolayers on 96-well plates were incubated with 1×10^−5^ to 10^−7^ M of nicotine tartrate for 48 h and stimulated with E44 cells (10^5^/ml at the beginning) for 2 h in EM. After incubation, monolayers were washed 4 times with PBS. Each well received 2×10^5^ PMN (0.2 ml) and was incubated for 90 min at 37°C. Then, cells were washed 5 times and fixed with 4% paraformaldehyde in PBS. Assays were performed in triplicate wells. Next, the mouse PMNs were stained with a mouse PMN-specific antibody against Ly-6G (Gr-1) IgG/FITC [56] and the numbers of PMN were counted under a fluorescence microscope. Fifteen microscope fields were randomly selected from 3 wells for each treatment to count the number of adherent leukocytes.

#### Immunofluorescence microscopy

MBMEC were grown in eight-well chamber slides coated with collagen. After treatment, MBMEC were washed with PBS and fixed with 4% paraformaldehyde or 95% ethanol (vol)–5%-acetic acid (vol) (for ZO-1) for 10-30 min at room temperature. After additional washes with PBS, MBMEC were blocked with 5% BSA in PBS for 30 min. Then, cells were stained with rhodamine-conjugated α-BTX and FITC-conjugated antibodies against GGT (rabbit), CD146 (mouse), S100B (rabbit) and ZO-1 [51]. The cells were then mounted with mounting medium containing DAPI (from Vector). Samples were examined under a Leica fluorescence microscope at the Congressman Dixon Cellular Imaging Core Facility, Children's Hospital Los Angeles. All pictures were taken using the same parameters to ensure that the fluorescence strength of each treatment could be compared and calculated.

#### Immunoblotting analysis

To assess protein expression in MBMEC, WT or KO cells were grown on 60 mm plates. Confluent MBMEC monolayers were incubated with 10 µM nicotine for different time points or different concentrations of nicotine (0.1–10 µM) for 48 h, stimulated with or without E44 (10^6^ CFU/ml) for 4 h. After completion of the incubation, total protein was extracted with SDS buffer, heated and subjected to SDS-polyacrylamide gel electrophoresis (SDS–PAGE) as described previously [23]. Total protein was transferred to nitrocellulose membranes by semi-dry blotting. After blocking with 5% milk in PBST (PBS containing 0.1% Tween20, Sigma) for 1 hour, membranes were probed with antibodies against α7 nAChR (rabbit Ab, 1 µg/ml, Genescript), ZO-1 (rabbit Ab, 2 µg/ml, Invitrogen), occludin (mouse Ab, 2 µg/ml, Invitrogen), and β-actin (rabbit Ab, 0.1 µg/ml, Santa Cruz Biotechnology,) for 2 h. The washed membranes were incubated with a HRP-conjugated secondary antibody for 1h and then visualized using an enhanced chemiluminescence procedure (Roche Applied Science, Indianapolis, IN).

### Transendothelial permeability assay

Transendothelial permeability assays were performed as described previously by measuring the passage of HRP through the confluent monolayer with transwell insert culture chambers [57]. Confluent WT and KO MBMEC monolayers on transwell inserts were exposed to 10 µM nicotine for 48 hours. After E44 stimulation (10^6^/ml within 0.2 mL) in the upper wells for 2 hours, the lower chamber was also refilled with fresh EM. Then, HRP (3 µg/ml) was added into each well. Twenty µl of EM was withdrawn from each lower chamber every hour. Ten µl was transferred to a 96-well ELISA plate, and the other 10 µl was diluted and plated on agar blood plate for bacteria number counting. After sample collection at different time points (0, 1, 2, 3, 4, 5 and 6 h), the experimental medium was subjected to the ELISA assay using TMB substrate (from KPL, Gaithersburg, MA). HRP activity was determined spectrophotometrically at 450 nm after adding the stop solution.

#### Mouse model of *E. coli* meningitis

Nicotine exposure was executed from day 8 to day 10 by feeding twice daily (free base 2.1 mg/kg body weight/day). For the study on chemical blockage of α7 nAChR, WT mice were exposed to nicotine (6–8 mice each group) and treated with or without the α7 antagonist MLA. MLA treatment started from day 8 to day 10 by intraperitoneal injection (10 mg/kg body weight) daily before the first nicotine exposure. Homozygous (α7^+/+^, α7^-/-^) and heterozygous (α7^+/-^) mice were exposed to nicotine as mentioned above. At 10 days of age, all pups received *E. coli* K1 strain E44 (2×10^5^ CFU) by intraperitoneal injection. Fifteen hours after *E. coli* inoculation, Evans blue (EB) was injected intraperitoneally (50 µg/g body weight). Three hours after receiving EB, animals were anaesthetized with ketamine and lidocaine, and blood samples were collected from heart puncture for bacterial culture using sheep blood plates. After perfusion from heart puncture with 20 ml PBS [58], the skull was opened. CSF samples were collected by washing the brain tissues with 100 µl of PBS, and then by washing the cerebral ventricles and cranial cavity with another 100 µl of PBS as described previously [27], [33]. CSF samples containing more than 10 erythrocytes per µl were discarded as contaminated samples [27], [33]. The brain tissues were cut into two halves. One half of the brain was put into a tube with 200 µl formamide to extract the EB. Subsequently, the optical density of the extracted EB was measured at 620 nm by spectrophotometry according to Zhang X *et al* [59]. The other half of the brain was mashed and diluted for bacterial culture with blood plates. For bacteria counting in CSF, 20 µl CSF samples were taken and diluted for bacterial culture with blood plates. For PMN counting in CSF, 50 µl CSF samples were stained with a FITC-conjugated rat anti-mouse Ly-6G (Gr-1) antibody and counted under fluorescence microscopy. Albumin concentrations in CSF samples were determined using a mouse Albumin ELISA kit from Bethyl laboratories (Montgomery, TX) according to the manufacturer. CSF samples were stored in −80°C for cytokine assays.

#### Mouse tobacco smoking exposure

Neonatal WT mice were divided into two groups (control and treatment, 7 mice each group). A TE-10 mouse smoke system with whole body exposure (Teague Enterprise, Davis, CA) was used with low tar research cigarettes (3R4F, Kentucky Tobacco Research& Development Center) [60] The mice at the age of 4 days were exposed to a mixture of main stream smoke (puffed smoke, 5%) and side stream smoke (smoke emitted by burning end of a cigarette, 95%) for 2 hours per day and a total of 7 days. Smoke particle concentration (TSP) in the chamber was maintained at 45±2 mg/m^3^. At 10 days of age, *E. coli* meningitis was induced as described above.

#### Histology immunostaining

Mouse brains were harvested 16 h after infection, fixed in 10% buffered formalin for 24 h, embedded in paraffin, and sections with 5 µm thickness were prepared. Tissue sections were stained with hematoxylin and eosin, and examined under a microscope to investigate histological alterations in the brain. Immunofluorescence staining of tight junction molecules occludin and ZO-1 was performed as described by Förster *et al* [61]. The prepared sections were incubated with antibodies against occludin (2 µg/ml) or ZO-1 (2 µg/ml) in 1% BSA at 4°C overnight, followed by a rhodamine-conjugated second antibody combined with a FITC conjugated mouse antibody against CD146 (1 µg/ml) in 1% BSA for 1 hour. Samples were washed with PBS and mounted with mounting medium containing DAPI (Vector Laboratories, Burlingame, CA). For α-BTX-staining of α7 nAChR in mouse brains, tissues sections were incubated with rhodamine-conjugated α-BTX for 1 hour, and mounted as described above. Photographs were taken under a Leica fluorescence microscope as described above. To examine neuron injury, the TUNEL assay was performed according to the manufacturer's protocol (Millipore, Chemicon, Billerica, MA). Then, the tissue sections were stained with a FITC-conjugated mouse antibody against neuron-specific nuclear protein (NeuN) (eBiosciences), and counterstained with DAPI. Image fluorescence quantification analysis was performed with program MetaMorph (Version 7.7.3.0) for tight junction molecules (ZO-1 and occludin) and TUNEL assays. For each treatment, 5-6 mouse brains were sectioned and stained.

#### Cytometric Bead Array (CBA) assay

The levels of cytokines in CSF, including IL-1β, IL-6, TNF-α, MCP-1 (CCL2), MIP-1α (CCL3), and RANTES (CCL5), were examined using the CBA assay (BD Biosciences, San Diego, CA) according to the manufacturer's protocol. Lyophilized protein (analyte) standards were multiplexed to contain a mixture of predetermined amounts of all analytes, and were used to prepare 9 serial dilutions, providing a range of concentrations from 10 to 2,500 pg/ml. Aliquots (50 µl each) of the analyte standards or experimental samples were mixed with 50 µl of premixed capture beads and incubated at room temperature (RT) for 1 h. Each set of capture beads is coated with a monoclonal antibody against a single analyte, and a mixture of 6 bead sets was combined to capture the 6 different analytes per sample. Next, we combined 6 PE-labeled detection antibodies against epitopes distinct from those recognized by the antibody-coated beads. Fifty microlitter of the mixed PE-labeled detection reagent was added to each sample and incubated for 2 h at RT in the dark. PE-conjugated detection antibodies stain beads proportionally to the amount of bound cytokine. Excess detection antibodies were removed by washing. The data were collected on an LSRII flow cytometer using DIVA software (BD Biosciences). FCAP software (BD Biosciences) was used to fit standard curves to the data obtained from the analyte standards and to calculate absolute concentration values for each of the 6 measured analytes from their respective standard curves.

#### Analysis of adhesion molecule expression *in vitro* and *in v*i*vo*

To assess the surface expression of adhesion molecules *in vitro*, α7^+/+^ and α7^-/-^ MBMEC were grown in 96-well plate, incubated with or without 10 µM nicotine for 48 h, and then stimulated with or without E44 (10^6^ CFU/ml) for 4 h. At the completion of incubation, cells were washed twice with PBS, fixed with 4% paraformaldehyde, and then blocked with PBS containing 5% BSA for 30 min. Cells were incubated with primary antibodies against ICAM-1 (rabbit Ab, 1 µg/ml, Abbiotec) and CD44 (mouse Ab, 1 µg/ml, Santa Cruz Biotechnology) at 4°C overnight. After washing, the cells were incubated with a HRP-conjugated secondary antibody for 1 h at room temperature. Liquid TMB substrate (KPL) was used for ELISA. For each assay, an isotype-matched control antibody was used in place of the primary antibody in three wells, and this background was subtracted from the signal. Analysis of adhesion molecules in CSF samples was performed by the same method using 96-well ELISA plates.

#### Measurements of intracellular [Ca^2+^]

To examine the role of α7 nAChR in *E. coli* induced calcium signaling, intracellular calcium flux in MBMEC was evaluated according to Kim KV *et al*. and Sukumaran SK *et al* [22], [62] with modifications. Briefly, MBMEC were cultured in Glass Bottom Culture Dishes (MatTek, Ashland, MA) in culture medium to 80% confluence with or without nicotine exposure (10 µM) and MLA incubation (1 µM) for 48 hours. Monolayers were washed with phenol-red-free HBSS and then incubated for 60 min with 4 µM Fura-2 AM and 0.04% Pluronic-127. Cells were then washed with phenol-red-free HBSS 2 times and incubated in this buffer for an additional 20 min. Then, cells were monitored for 10 min at 4 seconds intervals while recording the intensities of fluorescence at 340 nm and 380 nm. At the 2 min time point, E44 (1×10^8^ CFU) were added to stimulate MBMEC, and changes in intensities at 340 nm and 380 nm were measured. These Fura-2 AM experiments were performed on a Nikon Instrument Diaphot TMD 300 inverted microscope (Melville, NY), using a Nikon Fluor 40×/1.3 NA Ph4DL oil immersion objective lens. A Hamamatsu Corp. (Bridgewater, NJ) ORCA-100 (C4742–95-12NR) 12-bit digital camera was operated in 4×4 binning mode, with typical exposure times of 100–200 ms/channel. The microscope was equipped with a Ludl Electronics Products Ltd. (Hawthorne, NY) Mac2000 XYZ stage and a focus controller. The imaging rig was controlled by MetaMorph 4.5 (Universal Imaging Corp., Downingtown, PA). Changes in [Ca^2+^] were expressed as the F340:F380 ratio, where F340 and F380 were Fura-2 fluorescence intensities obtained at 340 nm and 380 nm excitation wavelengths, respectively.

#### Statistical analysis

For the analysis of the *in vitro* data, ANOVA and covariates followed by a multiple comparison test such as the Newmann-Keuls test were used to determine the statistical significance between the control and treatment groups. Software GraphPad Prsim 5.0 was used for analysis of data from animal experiments. P<0.05 was considered to be significant.

### Database

The protein access codes in Swissprot database are listed as follows: α7 nAChR, *Mus muscularus*, Q9JHD6; ZO-1, *Mus muscularus*, P39447; occludin, *Mus muscularus*, Q61146; CD44, *Mus muscularus*, P15379; ICAM-1, *Mus muscularus*, P13597; CD146, *Mus muscularus*, Q8R2Y2; S100B, *Mus muscularus*, P50114; GGT, *Mus muscularus*, Q60928; TNFα, *Mus muscularus*, P06804; IL-1β, *Mus muscularus*, P10749; IL-6, *Mus muscularus*, P08505; MCP-1, *Mus muscularus*, P10148; MIP-1α, *Mus muscularus*, P10855; RANTES, *Mus muscularus*, P30882.

## Supporting Information

Figure S1 **Isolation and characterization of WT and KO MBMEC.** **(A)** Images of MBMEC after isolation and purification using UEA-coated beads under light microscope (DIC). These cells (α7^+/+^ and α7^-/-^) were at passage 3. The WT and KO MBMEC were stained with FITC-conjugated antibodies against mouse CD146, GGT, S100B, and rhodamine-conjugated-α-BTX, respectively. The WT and KO MBMEC were also stained with a rabbit anti-ZO-1 Ab (FITC-conjugated) to show the formation of tight junctions. All bars are 25 µm. **(B)** Immunoblotting analysis of α7 nAChR from MBMEC (WT and KO). β-actin was used as an internal loading control. **(C)** WT and KO mouse brain cortex sections were stained with DAPI and rhodamine-conjugated α-BTX. Images are 100×. The squared areas were enlarged to show the details of α-BTX staining. Images are 200×.
(TIF)

Figure S2 **Effects of chemical and genetic blockages of α7 nAChR on pathogenicities of** ***E. coli*** **K1. (A-B)** Bacterial loads in the CSF of mice under different settings: (**A**) WT: Treatment with NT or MLA; and **(B)** WT and KO: Exposure to NT. **(C-D)** Flux of albumin into CSF of mice under different settings: (**C**) WT: Treatment with NT or MLA; and **(D)** WT and KO: Exposure to NT (n = 6–8). WT mice without treatment (NT or MLA) served as the controls *P<0.05, **P<0.01.
(TIF)

Figure S3 **Nicotine increased pathogenicities of** ***E. coli*** **K1 in WT and heterozygous (HZ) (+/-) mice.** *E. coli* meningitis was induced in neonatal mice under 4 different settings (n = 6–7) (I: WT; II: WT+NT; III: HZ; IV: HZ+NT). **(A)** Bacteremia; **(B)** Bacterial loads in the brains; **(C)** Bacterial loads in the CSF; **(D)** Recruitment of PMN into the CSF; and **(E)** Flux of albumin into the CNS. *P<0.05, **P<0.01.
(TIF)

Figure S4 **Tobacco smoking (TS) increased pathogenicities of** ***E. coli*** **K1 in the neonatal meningitis model.** *E. coli* meningitis was induced in neonatal mice under two different settings (n = 7) [I: WT (Control); II: WT+TS]. **(A)** Bacteremia; **(B)** Bacterial loads in the brains; **(C)** Bacterial loads in the CSF; **(D)** Recruitment of PMN into the CSF; and **(E)** Flux of albumin into the CNS. *P<0.05, **P<0.01.
(TIF)

Figure S5 **Effects of blockages of α7 nAChR on NT- and E44-induced tight junction (TJ) disruption**. (**A-C**) Immunoblotting analysis of occludin, ZO-1 and α7 nAChR under different experimental settings: (**A**) WT MBMEC +NT (0.1–10 µM for 48 h); **(B)** WT MBMEC+NT (10 µM for 0-72h); **(C)** WT MBMEC+NT (10 µM for 48h)+MLA (0–10 µM for 48 h). In **(A-C)**, β-actin was used as an internal loading control. **(D)** Fluorescence-based quantification of ZO-1 expression in WT and KO mouse brain cortex with or without NT exposure upon E44 infection (n = 5–6). The WT mouse control without any treatment was taken as one fold. (**E**) Immunostaining of TJ molecules in mouse brain cortex with or without NT exposure under different settings (I: CON: No treatment; II. E44; III: E44+NT). The tissue section was stained with antibodies against ZO-1 (rhodamine-conjugated) and CD146 (FITC-conjugated). DAPI staining was used to show the structures of brain cortex. Images are 200×.
(TIF)
