# Supplementary figures and images for: Meningitic Escherichia coli K1 Penetration and Neutrophil Transmigration Across the Blood–Brain Barrier are Modulated by Alpha7 Nicotinic Receptor
Source: PLoS One. 2011 Sep 22;6(9):e25016. doi: 10.1371/journal.pone.0025016 (PMC3178609; doi:10.1371/journal.pone.0025016)

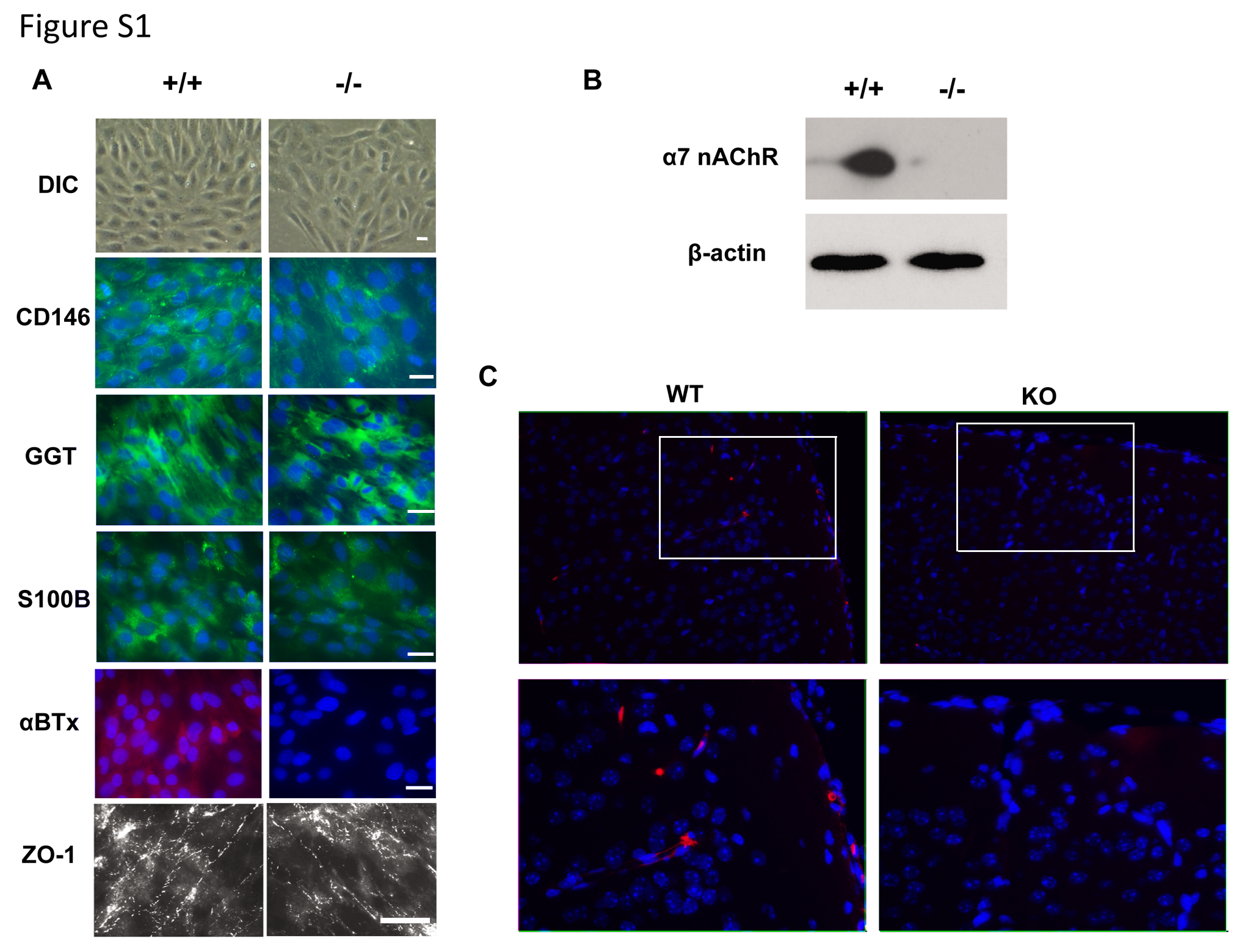

Supplement: Figure S1 — Isolation and characterization of WT and KO MBMEC. (A) Images of MBMEC after isolation and purification using UEA-coated beads under light microscope (DIC). These cells (α7+/+ and α7-/-) were at passage 3. The WT and KO MBMEC were stained with FITC-conjugated antibodies against mouse CD146, GGT, S100B, and rhodamine-conjugated-α-BTX, respectively. The WT and KO MBMEC were also stained with a rabbit anti-ZO-1 Ab (FITC-conjugated) to show the formation of tight junctions. All bars are 25 µm. (B) Immunoblotting analysis of α7 nAChR from MBMEC (WT and KO). β-actin was used as an internal loading control. (C) WT and KO mouse brain cortex sections were stained with DAPI and rhodamine-conjugated α-BTX. Images are 100×. The squared areas were enlarged to show the details of α-BTX staining. Images are 200×. (TIF) [file pone.0025016.s001.tif]

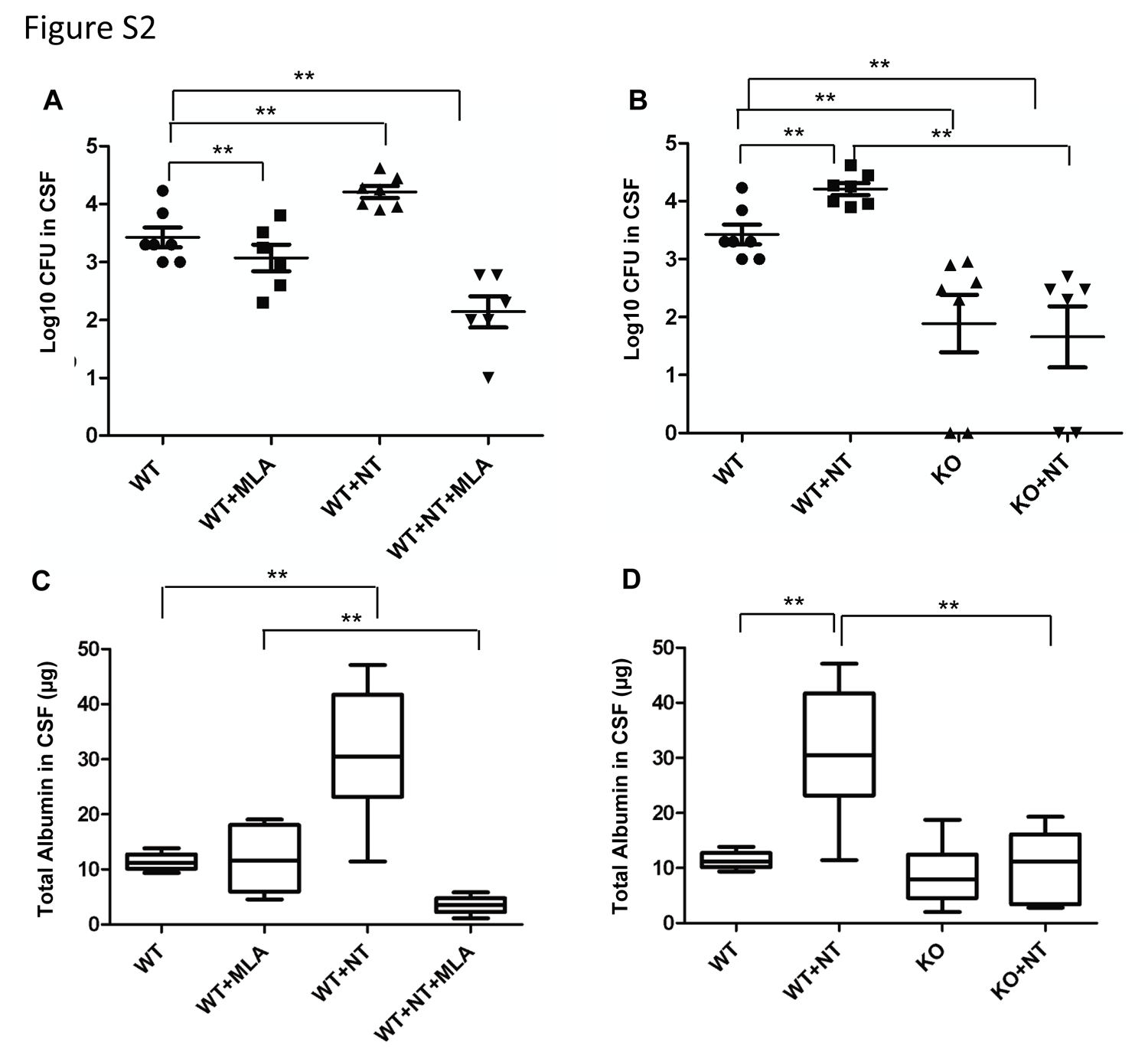

Supplement: Figure S2 — Effects of chemical and genetic blockages of α7 nAChR on pathogenicities of E. coli K1. (A-B) Bacterial loads in the CSF of mice under different settings: (A) WT: Treatment with NT or MLA; and (B) WT and KO: Exposure to NT. (C-D) Flux of albumin into CSF of mice under different settings: (C) WT: Treatment with NT or MLA; and (D) WT and KO: Exposure to NT (n = 6–8). WT mice without treatment (NT or MLA) served as the controls *P<0.05, **P<0.01. (TIF) [file pone.0025016.s002.tif]

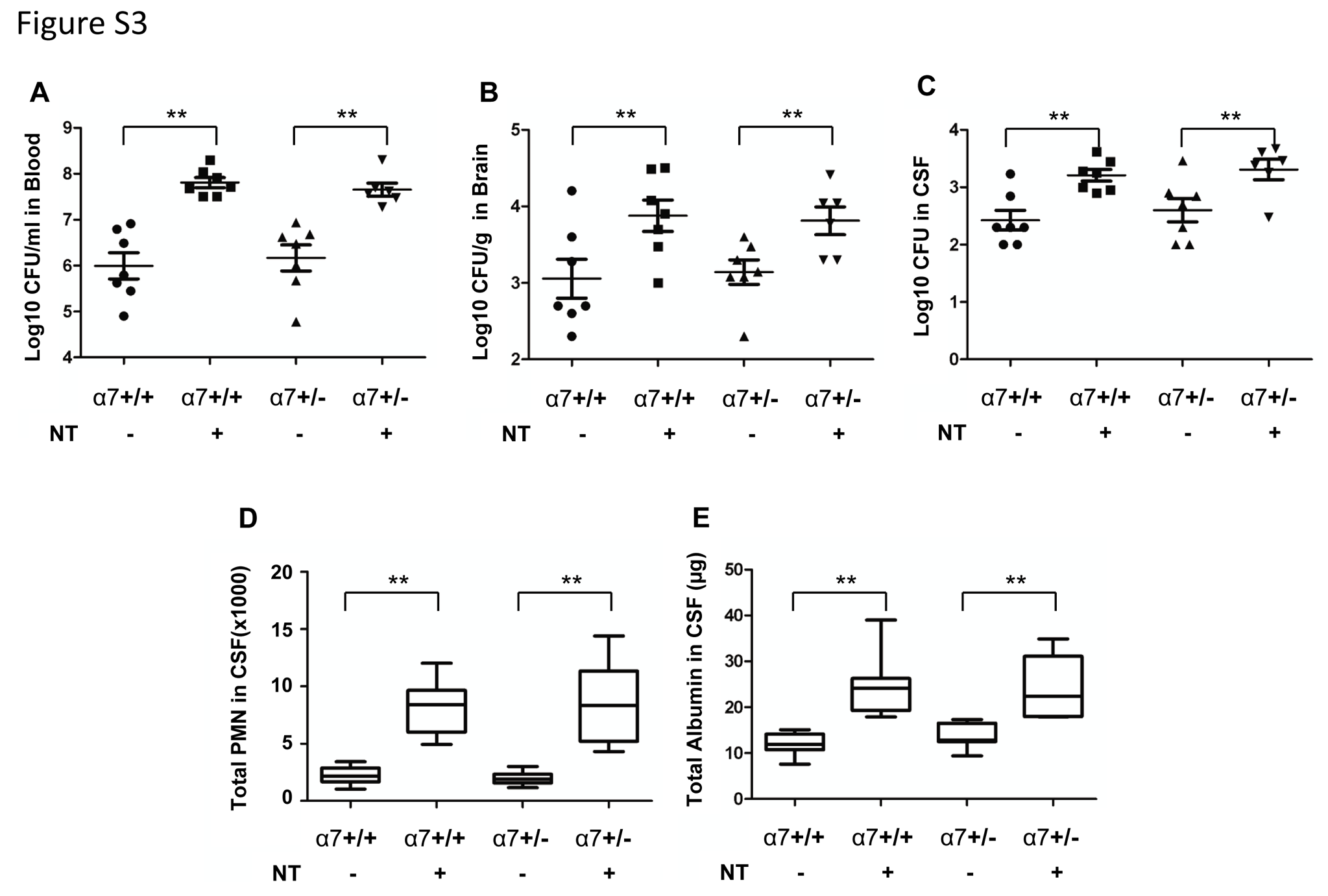

Supplement: Figure S3 — Nicotine increased pathogenicities of E. coli K1 in WT and heterozygous (HZ) (+/-) mice. E. coli meningitis was induced in neonatal mice under 4 different settings (n = 6–7) (I: WT; II: WT+NT; III: HZ; IV: HZ+NT). (A) Bacteremia; (B) Bacterial loads in the brains; (C) Bacterial loads in the CSF; (D) Recruitment of PMN into the CSF; and (E) Flux of albumin into the CNS. *P<0.05, **P<0.01. (TIF) [file pone.0025016.s003.tif]

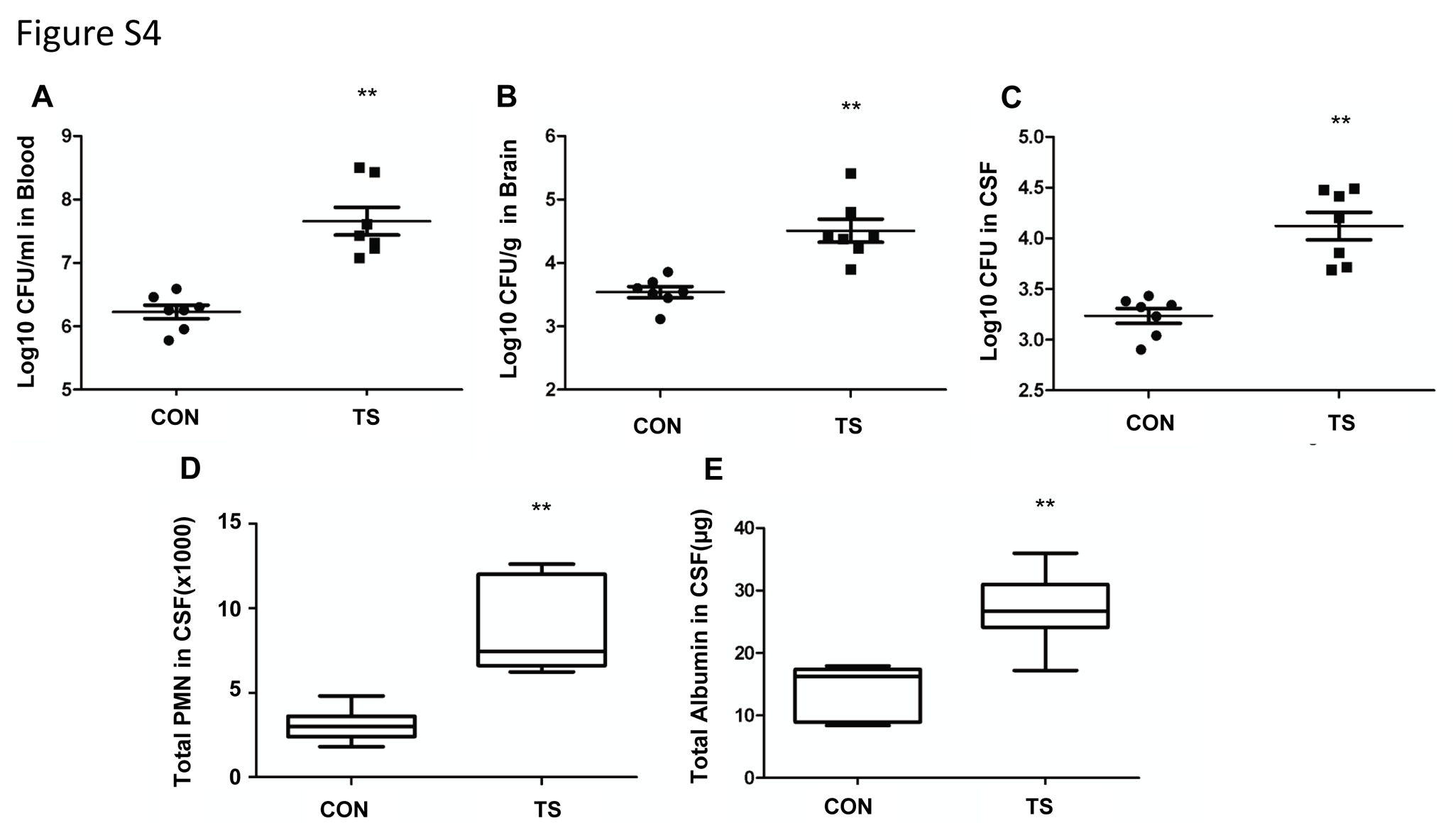

Supplement: Figure S4 — Tobacco smoking (TS) increased pathogenicities of E. coli K1 in the neonatal meningitis model. E. coli meningitis was induced in neonatal mice under two different settings (n = 7) [I: WT (Control); II: WT+TS]. (A) Bacteremia; (B) Bacterial loads in the brains; (C) Bacterial loads in the CSF; (D) Recruitment of PMN into the CSF; and (E) Flux of albumin into the CNS. *P<0.05, **P<0.01. (TIF) [file pone.0025016.s004.tif]

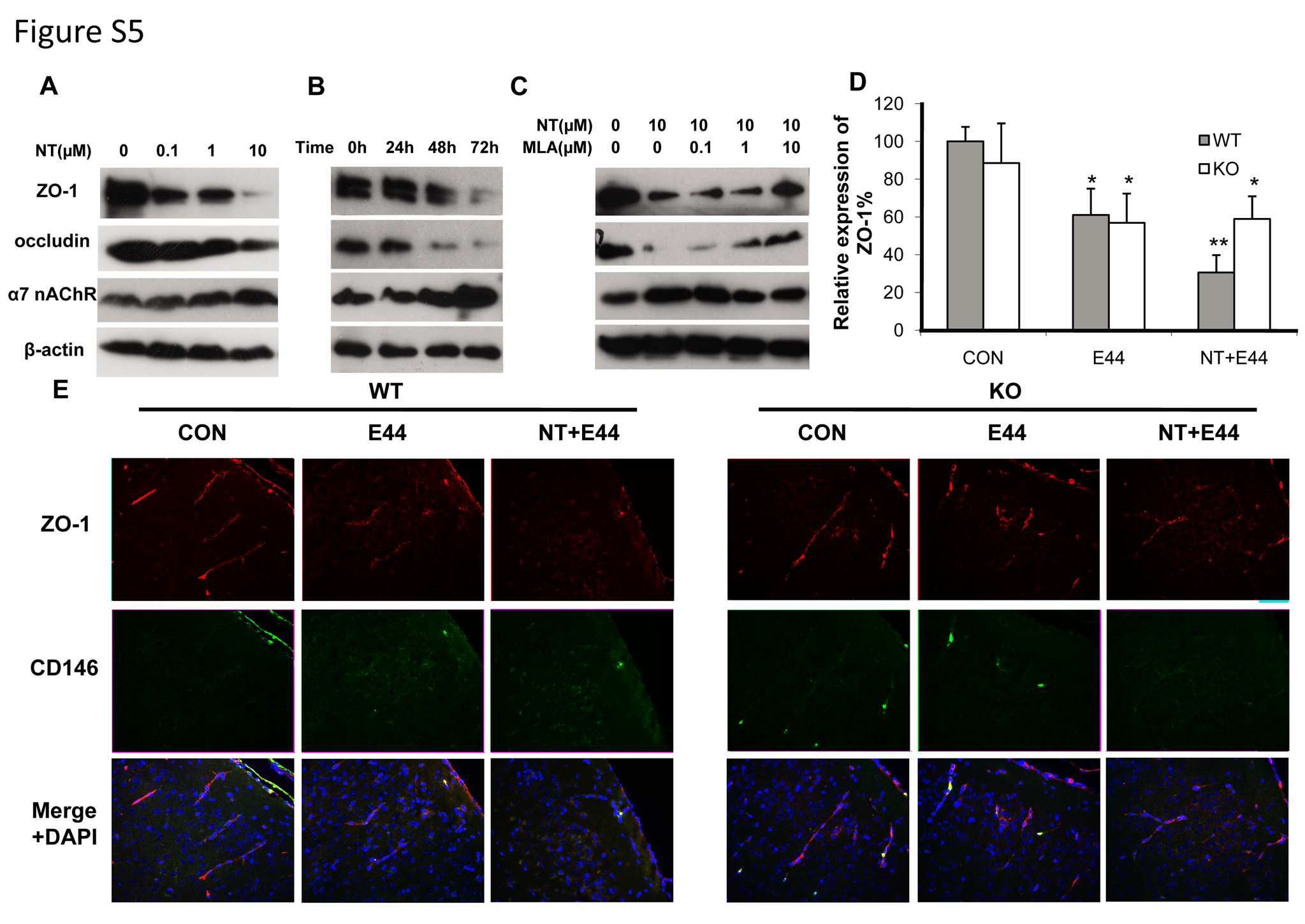

Supplement: Figure S5 — Effects of blockages of α7 nAChR on NT- and E44-induced tight junction (TJ) disruption. (A-C) Immunoblotting analysis of occludin, ZO-1 and α7 nAChR under different experimental settings: (A) WT MBMEC +NT (0.1–10 µM for 48 h); (B) WT MBMEC+NT (10 µM for 0-72h); (C) WT MBMEC+NT (10 µM for 48h)+MLA (0–10 µM for 48 h). In (A-C), β-actin was used as an internal loading control. (D) Fluorescence-based quantification of ZO-1 expression in WT and KO mouse brain cortex with or without NT exposure upon E44 infection (n = 5–6). The WT mouse control without any treatment was taken as one fold. (E) Immunostaining of TJ molecules in mouse brain cortex with or without NT exposure under different settings (I: CON: No treatment; II. E44; III: E44+NT). The tissue section was stained with antibodies against ZO-1 (rhodamine-conjugated) and CD146 (FITC-conjugated). DAPI staining was used to show the structures of brain cortex. Images are 200×. (TIF) [file pone.0025016.s005.tif]
